# Research progress of EMOFs-based burning rate catalysts for solid propellants

**DOI:** 10.3389/fchem.2022.1032163

**Published:** 2022-10-13

**Authors:** Bojun Tan, Xiong Yang, Jinkang Dou, Binghui Duan, Xianming Lu, Ning Liu

**Affiliations:** Xi’an Modern Chemistry Research Institute, Xi’an, Shaanxi, China

**Keywords:** solid propellant, energetic metal organic frameworks (EMOFs), energetic burning rate catalysts, environmental protection, high energy and low sensitivity, nanometerization, multifunctional compounding

## Abstract

Energetic Metal Organic Frameworks (EMOFs) have been a hotspot of research on solid propellants in recent years. In this paper, research on the application of EMOFs-based burning rate catalysts in solid propellants was reviewed and the development trend of these catalysts was explored. The catalysts analyzed included monometallic organic frameworks-based energetic burning rate catalysts, bimetallic multifunctional energetic burning rate catalysts, carbon-supported EMOFs burning rate catalysts, and catalysts that can be used in conjunction with EMOFs. The review suggest that monometallic organic frameworks-based burning rate catalysts have relatively simple catalytic effects, and adding metal salts can improve their catalytic effect. Bimetallic multifunctional energetic burning rate catalysts have excellent catalytic performance and the potential for broad application. The investigation of carbon-supported EMOFs burning rate catalysts is still at a preliminary stage, but their preparation and application have become a research focus in the burning rate catalyst field. The application of catalysts that can be compounded with EMOFs should be promoted. Finally, environmental protection, high energy and low sensitivity, nanometerization, multifunctional compounding and solvent-free are proposed as key directions of future research. This study aims to provide a reference for the application of energetic organic burning rate catalysts in solid propellants.

## 1 Introduction

As the power source of solid rocket motors, solid propellants play an important role in the development of missile and space technology. Combustion characteristics are a critical factor affecting the performance of solid propellants, including the energy release rate, efficiency and stability. Combustion characteristics are closely related to the combat effectiveness of missile weapons. Burning rate catalysts are the functional material that adjusts the combustion characteristics of solid propellants (Wang et al., 2015; Zhao F. et al., 2016; Vake et al., 2021; Zhang et al., 2021). Burning rate catalyst refers to a kind of functional materials that regulate the combustion process of solid propellants and control the sensitivity of the combustion characteristics of solid propellants to environmental factors such as pressure and temperature by influencing the gas-solid phase reaction process in the combustion process of solid propellants, so as to achieve reliable and stable combustion of solid propellants. Therefore, researchers have been devoted to developing new high-efficiency burning rate catalysts, broadening the burning rate range of solid propellants and reducing the pressure index. At present, non-energetic inorganic or organic lead-copper salts are used as burning rate catalysts for most double-base solid propellants. However, these salts reduce the energy level of solid propellants to a certain extent. Energetic Metal Organic Frameworks (EMOFs) have a large specific surface area, a regular pore structure and highly dispersed active sites on the surface. Compared to traditional burning rate catalysts, EMOFs-based catalysts can tremendously increase the energy level of solid propellants. Moreover, EMOFs can improve the specific impulse of the propellant system and expand the development of high-energy solid propellants (Zhang and Shreeve, 2016; Dalinger et al., 2021; Qin et al., 2022). EMOFs-based burning rate catalysts have two advantages. On the one hand, rational design or modification of organic energetic ligands, which are the source of energy in EMOFs-based burning rate catalysts, can ensure the high energy level of the system. On the other hand, the metal ions in EMOFs can not only adjust and control the sensitivity of the frameworks system, but also generate metal oxides *in situ* to regulate combustion properties of solid propellants during the combustion process. In addition, the metal (metal oxide)-carbon-supported composites formed *in situ* during the catalytic combustion process of EMOFs may also be efficient burning rate catalysts. These characteristics, which are different from those of previous catalysts, make EMOFs-based materials a potential choice of catalysts for high efficiency solid propellants and an ideal tool to study the structure-activity relationship of burning rate catalysts. Therefore, EMOFs-based burning rate catalysts have received extensive attention of researchers engaged in material synthesis and solid propellant formulation in recent years. In this paper, to facilitate the development of energetic burning rate catalysts that are urgently needed, the research status of four types of energetic burning rate catalysts for solid propellants was reviewed. The energetic burning rate catalysts studied included monometallic organic frameworks-based catalysts, bimetallic multifunctional type catalysts, carbon-supported EMOFs-based catalysts, and catalysts that can be used in conjunction with EMOFs. Besides, the development prospect of these catalysts was discussed. This paper provides reference for research on organic energetic burning rate catalysts applied in solid propellants.

## 2 Monometallic organic frameworks-based energetic burning rate catalysts

Monometallic organic frameworks-based energetic burning rate catalysts are a class of EMOFs-based burning rate catalyst. A great majority of the EMOFs reported in literature are monometallic ones, and their catalytic mechanism has been expounded.

### 2.1 Five-membered nitrogen heterocyclic ligand-based burning rate catalysts

#### 2.1.1 Azole-containing metal organic frameworks-based burning rate catalysts

The most widely studied azoles in literature are tri/tetrazole compounds, which are five-membered nitrogen-containing heterocyclic compounds. The nitrogen atoms on the ring can provide abundant coordination sites (Zhang J. et al., 2017). They can participate in coordination as neutral molecules, or lose protons and become anions to balance the charge. At the same time, nitrogen atoms can also accept protons to form hydrogen bonds, thereby reducing the mechanical sensitivity and improving stability of complexes. In addition, the tri/tetrazole ring has good thermodynamic stability and high enthalpy of formation due to the high content of C–N, N–N and N=N bonds (Hiskey et al., 1998; Thomas and Caries, 2008).

**FIGURE 1:**
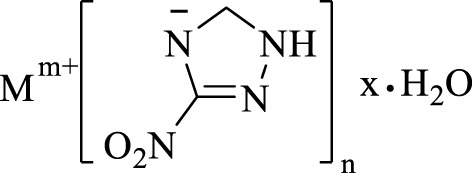
Chemical formula of NTO metallic salts (Lee et al., 1987).

3-nitro-1,2,4-triazol-5-one (NTO) metal salts are a currently mature azole-containing metal organic frameworks-based burning rate catalyst. NTO is a highly polar triazole-type high-energy molecule, which contains multiple coordination atoms, such as nitro oxygen atoms, carbonyl oxygen atoms, ring-forming nitrogen atoms, *etc.* These atoms are apt to form complexes and coordinate with metal ions. The most commonly used conventional energetic burning rate catalysts are NTO-containing metal organic frameworks-based burning rate catalysts, which are prepared by adding NTO-containing energetic organic ligands a Lewis base to a metal salt. The biggest advantage of this type of catalyst is its simple and efficient preparation. This catalyst can be prepared in large scale, and its preparation is less affected by experimental conditions. In addition, with high-energy, heat-resistant, dense and insensitive properties, NTO-containing metal organic frameworks-based burning rate catalysts can not only greatly improve the burning rate and specific impulse of propellants, but also reduce the pressure index (Figure 1). Therefore, NTO-containing metal organic frameworks-based burning rate catalysts have a wide application (Lee et al.,1987; Lee and Coburn, 1988).

In 1987, K. Y. Lee (Lee et al.,1987) carried out the synthesis and characterization of 3-nitro-1,2,4-triazol-5-one (NTO), and the study showed that the material has compact structure and high enthalpy, which is a high-energy and insensitive energetic material. In 1991, X. Y. Fu and others (Li et al., 1993) synthesized lead salts, copper salts and iron salts of NTO. The structures of the three metal salts were preliminarily determined by NMR, infrared and elemental analysis, and their physical and chemical properties were also preliminarily studied. But their crystal structures could not be obtained. In 1993, S. W. Li et al. (Guan et al., 1999) studied the catalytic properties of NTO lead and copper salts as energetic burning rate catalysts in GAP propellants and modified double-base propellants. The burning catalytic performances of these two salts were close to that of lead aromatic acid and copper salt, which can be used as high-pressure platform burning catalyst. The catalysts can also increase the detonation heat of the formula by 47.7 kJ kg^−1^, which is beneficial to the improvement of energy. Since 1993, T. L. Zhang (Zhang et al., 1993; Zhang et al., 1994; Zhang et al., 1998; Zhang et al., 2002) and J. R. Song (Song et al., 1996; Chen et al., 1997; Song et al., 1997; Chen et al., 1998; Song et al., 1998; Song et al., 2003) synthesized 26 kinds of metal salts of NTO, and focused on the crystal structure and chemical structure of NTO copper salt, lead salt, *etc.* They also preliminarily studied their thermal decomposition mechanisms. These studies have laid a solid foundation on the application of NTO salts as energetic burning rate catalysts. In 1999, D. L. Guan (Guan et al., 1999) prepared NTO barium salt (Ba(NTO)·3H_2_O) which has the characteristics of “green” energetic burning rate catalyst (i.e., easy-to-obtain, non-toxic and safe), in view of the toxicity of NTO lead salt as a burning rate catalyst. Its application in composite propellants was preliminarily explored. The thermal decomposition was weakened when the pressure was increased. It is a potential “green” energetic burning rate catalyst with low characteristic signal.

In 2002, Singh et al. (Singh and Felix, 2002) reviewed more than 50 NTO salts and classified them into alkaline earth metal salts, alkali metal salts, rare metal salts, transition metal salts, metal complexes, aromatic amine salts and aliphatic amine salts. At the same time, their performances were evaluated, and it is pointed out that the NTO transition metal salts have the advantages of high energy, low sensitivity and stability. In 2003, Singh et al. (Singh and Felix, 2003) synthesized a series of NTO transition metal salts. They found that Cu(NTO)_2_ had the best catalytic effect on HTPB-PA solid composite propellant, while Zn(NTO)_2_ had the best catalytic effect on HTPB-AN solid composite propellant. In 2007, X. Z. Fan et al. (2007) showed that NTO lead and copper salts can both promote the decomposition of NC/NG in the AP-CMDB propellant system, so that the burning rate of AP-CMDB propellant is increased under the lower pressure range of 1–7 MPa. Moreover, the burning rate is decreased under the high pressure of 10–20 MPa, and the burning rate pressure index of the propellant is reduced. In 2010, J. H. Yi et al. (2010) synthesized 3-nitro-1,2,4-triazol-5-one bismuth (Bi-NTO) metal salt burning rate catalyst. The results of TG-DTG and DSC tests showed that the complex can significantly increase the burning rate and reduce the pressure index of the NG/TEGDN/NC propellant, and is a “green” energetic burning rate catalyst. In 2013, X. P. Huang et al. (2013) aimed to solve the problem that NTO acid lead salts contain acidic protons, which seriously limited their application in propellants. Through “one-pot” neutralization and metathesis reaction, the separation yield was 99.5% for the first time. NTO lead ortho-salt NP (NTO-Pb) was synthesized, and its physicochemical properties were preliminarily tested to provide reference for its application in propellants.

NTO-containing metal organic frameworks have been the most mature energetic burning rate catalysts in recent years. Their synthesis, properties, and thermal decomposition mechanisms have been thoroughly studied. They can improve the burning rate and specific impulse of the formula system, and reduce the pressure index. In addition, there are hydrogen bonds formed in complexes constituted by NTO^−^ with strong polarity and metal ions. Hydrogen bonds facilitate the transfer of excess electrons on oxygen and nitrogen, render the system more insensitivity, and thereby reduce the sensitivity. Therefore, NTO-containing metal complexes have high energy and low sensitivity, showing broad application prospects (Table 1).

**TABLE 1:** The physicochemical properties of some NTO salts.

Parameters	NTO	Pb-NTO	Cu-NTO	Ba-NTO	Pb(NTO)_2_	PDNI	NH_4_-NTO
melting point/(°C)	240	208	>260	215	241	-	198–200
density/(g·cm^−3^)	1.93	2.939	-	2.83	4.24	-	1.68
Impact sensitivity/(J)	>280	0	0	0	68	0	0
Friction sensitivity/(N)	>353	60	0	0	100	>360	0
Decomposition temperature	273	217.8	268.8	216.8	246.7	279	279
Ref	Rothgery et al. (1991)	Li et al. (1993)	Li et al. (1993)	Guan et al. (1999)	Huang et al. (2013)	Zheng et al. (2006)	Guan et al. (1999)

T. M. Klapötke et al. (Klapötke and Rienacker, 2001) studied organic azide metal salts as burning rate catalysts, and found that organic azide metal salts are safer than lead azide or silver azide salts as energetic burning rate catalysts under the same conditions. Organic azide metal salts were better energetic burning rate catalysts from the perspective of energy, but their sensitivity was still higher than other types of catalysts. As a result, they are not suitable for use as propellant components, which greatly limits their use as burning rate catalysts in solid propellants. In contrast, the chemical properties of tetrazoles were similar to those of azides, but their mechanical sensitivity was much lower. So their performance is more stable.

In 1988, Bates (Bates, 1988) published a review article on the application of tetrazole compounds in priming systems, expounding the broad application prospects of such compounds in the field of energetic materials. In 2000, Y. C. Lu et al. (Lu and Wierenga, 2000)carried out three investigations on tetrazole compounds as propellant burning rate catalysts. At 7 MPa, all three tetrazole compounds could make the solid propellants burning rate greater than 63.5 mm s^−1^. When the combustion pressure was 7–19 MPa, the pressure index of the propellant containing 5-ATZ/BTATz was 0.7.

**FIGURE 2:**
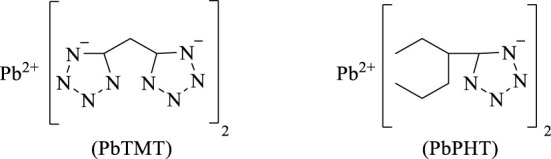
5-dimethytetrazole lead salt (PbTMT) and phenytetrazole lead salt (PbPHT) (Zhao et al., 2004).

In 2003, M. Z. Deng et al. (2003) prepared lead, copper and strontium salts of 5-phenyltetrazole and 5-methylenebistetrazole, and explored the application of these three metal salts as catalysts in composite propellant formulations. The results showed that the burning rates of these two tetrazolium lead salts were higher than the basic formula in the range of the measured pressure, showing good catalytic performance. In 2004, F. Q. Zhao et al. (2004) studied the effect of lead, copper and strontium salts of tetrazolium compounds (Figure 2) on the combustion performance of the composite modified containing hexogen double-base propellant (RDX-CMDB), and found that 5-dimethylene lead tetrazolium (PbTMT) and lead phenyltetrazolium (PbPHT) had good catalytic effects and potential advantages in applications such as novel low-signal propellants.

In 2005, M. Friedrich et al. (2005) successfully synthesized three aminobistetrazole divalent copper complexes, including Cu(bta) (NH_3_)_2_, Cu(bta) (NH_3_)_2_·H_2_O and (NH_4_)_2_Cu(bta)_2_.2.5H_2_O. The results showed that the three complexes had good thermal stability, higher enthalpy and suitable sensitivity. When mixed with solid propellant, it was found that they had a good catalytic effect, which can effectively reduce the thermal decomposition temperature of propellant components. They were expected to become green burning rate catalysts.

In 2007, J. Li et al. (2007) synthesized the first azido-metal-triazolate coordination complexes by a hydrothermal method, the complex had a rare three-dimensional non-interpenetrated utp or (10,3)-d topological networkstructure and exhibited spin-canted antiferromagnetism at low temperature. In 2016, J. Li et al. (Zhao H. et al., 2016) continued synthesized four new azido-Cu(II) complexes with mono-*N*-donor pyridine based co-ligands to use low-temperature hydrothermal reactions of copper salts with NaN_3._ In addition, the co-ligands in three were generated *in situ* by different reactions of pyridine derivatives. And the *in situ* reactions are clearly vital in obtaining the three complexes. It was also found that the uncoordinated groups in the co-ligands play a key role in determining the structures and finally the magnetism of the four complexes. This work demonstrateed that it was a powerful approach to construct magnetic metal-azide complexes with unique structures and varied magnetism by using a co-ligand generated *in situ* in hydrothermal reactions [Cu_3_(pyridine)_2_(N_3_)_6_]_*n*_. Although the authors did not estimate the detonation performance of these kinds of EMOFs, the density of [Cu_3_(Pyridine)_2_(N_3_)_6_]_*n*_ compounds was as high as 2.0 g cm^−3^, so it could be predicted that these kinds of EMOFs have relatively excellent detonation performance and is a potential burning rate catalysts.

In 2009, T. M. Klapötke et al. (Klapötke et al., 2009a; Klapötke et al., 2009b; Hartdegen et al., 2009; Karaghiosoff et al., 2009) carried out a lot of work on the synthesis and application of tetrazole-containing energetic complexes, and prepared alkali metal, alkaline earth metal and transition metal salts of 5-carboxytetrazole, 1-ethyl-5-nitrotetrazoleand 5-nitrotetrazole. They found that the tetrazole metal salts had high energy content and good stability, most of which were insensitive to impact and friction. The main frame of some compounds remained stable under 380°C, indicating that tetrazolium-based metal salts have potential values as heat-resistant high-energy and insensitive burning-rate catalysts. The above reported results showed that tetrazolium metal salts had the advantages of high energy, compact structure, high enthalpy, good stabilityand high catalytic activity. As a result, they have considerable application prospects as energetic burning rate catalysts in various propellants. The preparation of novel high-energy and low-signature EMOFs as burning rate catalysts from tetrazole-based high-nitrogen energetic ligands and metal salts will become the preferred route to increase the energy level of solid propellants and improve their combustion performance.

In 2009, Q. Yang et al. (Yang., 2019) used solution method and hydrothermal/solvothermal method to introduce carboxylic acid compounds into the synthesis of Cu, Co, Ni/triazole and tetrazole transition metal energetic complexes. Thirty kinds of complexes that have not been reported in the literature were obtained, and all of the obtained complexes had different effects on advancing decomposition peak temperature, increasing heat release and decreasing activation energy for double-base solid propellants. In addition, a main rule can be found from the catalysis of different complexes on the same single-component system of propellants that the influence of Cu^2+^ complexes was greater than that of Co^2+^ and Ni^2+^ complexes. It is worth noting that the carboxylic acid can play a role in adjusting the oxygen balance of the propellant components. The experimental results also showed that the increase of the oxygen content of the complex had an obvious effect on the thermal decomposition of the mixed system.

In 2011, Z. Tang et al. (2011) mixed tetrazolium acetic acid (Htza) with basic copper carbonate to obtain the energetic complex [Cu(tza)_2_]_n_, studied its thermal stability and the catalytic effect on the pyrolysis of cyclotrimethyltrinitroamine (RDX). The results show that the compound not only had high thermal stability, but also had a good catalytic effect on RDX, which can advance the decomposition peak temperature of RDX by 16.7°C.

In 2013, Q. Yang et al. (2013) used 3,5-diamino-1,2,4-triazole (Hdatrz) as the main ligand and fumaric acid (H_2_fma) as the auxiliary ligand to obtain [Ni_3_(Hdatrz)_6_ (fma)_2_(H_2_O)_4_]fma complex by solid phase synthesis. It has been found that the complex had a good catalytic effect on the thermal decomposition of the oxidant ammonium perchlorate (AP) in the hydroxy-terminated polybutadiene (HTPB) propellant.

In 2014, W. Gao et al. (2014) used lead nitrate to perform coordination reaction with the energetic ligand 5-tetrazolyl-1,2,4-triazole (H_2_tztr) to obtain two energetic complexes [Pb(Htztr)_2_(H_2_O)]_n_ and [Pb(H_2_tztr) (O)]_n_. Among them [Pb(H_2_tztr) (O)]_n_ was an energetic material with excellent performance, and had higher density and better detonation performance than [Pb(Htztr)_2_(H_2_O)]_n_. The density of [Pb(H_2_tztr) (O)]_n_ was as high as 3.511 g cm^−3^, the detonation velocity was 8,122 m s^−1^, and the detonation pressure was 40.12 GPa. Catalytic pyrolysis experiments found that [Pb(Htztr)_2_(H_2_O)]_n_ can combine the two exothermic peaks of pure AP into one violent exothermic peak and reduce the exothermic peak temperature by about 19°C. In addition [Pb(H_2_tztr) (O)]_n_ can advance the exothermic peak temperature of AP by about 18°C, and [Pb(Htztr)_2_(H_2_O)]_n_ and [Pb(H_2_tztr) (O)]_n_ can reduce the exothermic peaks of RDX by 16°C and 11°C, respectively.

In 2014, N. Li et al (2014) obtained Cu(AT)_4_H_2_O(ClO_4_)_2_ (ATCP) by coordinating 4-amino-1,2,4-triazole (AT) with Cu(ClO_4_)_2_. The modified double-base propellant samples containing ATCP were prepared by casting process, and the effect of ATCP on the combustion performance of the propellant was studied. DSC test results showed that ATCP was an excellent burning catalyst, which can significantly accelerate the burning rate of the propellant. The results showed that the increase in the burning rate of the modified double-base propellant was proportional to the mass fraction of ATCP at 11–18 MPa. ATCP can increase the burning rate of the modified double-base propellant by 85%, the speed can be increased from 26.41 mm s^−1^–48.79 mm s^−1^ at 18 MPa.

In 2015, D. Chen et al. (2015) synthesized two examples of Ni complexes based on 5,5-azotetrazole ligands [Ni(en)_3_](AZT)THF(en, ethylenediamine; THF, tetrahydrofuran) and [Ni(AZT) (pn)_2_](pn, propylenediamine), and the effects of these two compounds on the thermal decomposition process of RDX, HMX and AP were tested. The results showed that the two energetic complexes can accelerate the thermal decomposition of RDX, HMX and AP, in which the complex [Ni(AZT) (pn)_2_] was more effective than [Ni(en)_3_](AZT)THF for RDX and HMX. It was worth mentioning that the catalytic effect of [Ni(en)_3_](AZT)THF, Ni(en)_3_SO_4_ and SAZT were compared and the result showed that the catalytic effect of [Ni(en)_3_](AZT)THF was superior. This catalytic effect was attributed to the decomposition and exotherm of the complex itself, because [Ni(en)_3_](AZT)THF contains energetic ligand AZTZ^−^ which can provide heat by decomposition itself, which will advance the thermal decomposition temperature of metal ion Ni^2+^. This synergy between energetic ligands and metal ions is prevalent in EMOFs.

In 2016, K. Li et al. (2016) synthesized two energetic complexes using the energetic ligand tetrazolium acetate ({Bi(tza) (C_2_O_4_) (H_2_O)}·H_2_O)_n_ and [Fe_3_O(tza)·6(H_2_O)_3_]NO_3_. The anion tza^−^ and cation {Bi(C_2_O_4_)}^+^ in the metal Bi complex were connected to each other to form a three-dimensional columnar layered structure, and the metal Fe complex was a zero-dimensional structure composed of NO_3_^−^ and the cation cluster [Fe_3_O(tza)_6_]^+^. The authors used TG-DSC to test the effect of the two complexes on the thermal decomposition of AP. The results showed that both metallic Fe and Bi complexes exhibited good catalytic effects on AP, but the Fe complex had a slightly better catalytic effect. It was worth mentioning that if the Fe and Bi complexes are mixed at a molar ratio of 1:2, the effect is more significant than that of the single catalyst. In 2017, K. Li et al. (Qu et al., 2017) continued to use tetrazolium acetate as the ligand, and synthesized [Co(tza)_2_]_n_,{[Cu_4_(tza)_6_(OH)_2_·H_2_O}_n_ [Mn (tza)_2_]_n_ and {[Bi(tza) (C_2_O_4_) (H_2_O)]·H_2_O} They then studied their influence on the thermal decomposition of HMX. Experiments showed that the synthesized complexes had obvious catalytic effect on HMX, and the Cu complex had the best catalytic effect, indicating that the catalytic effect was closely related to the oxides produced by thermal decomposition. The authors also investigated the catalytic effect of different mixed samples on the thermal decomposition of HMX. The catalytic performances are in the order of Cu-Mn system > Fe-Bi system > Co-Bi system. The authors speculated that the catalytic effect was attributed to the oxides generated by thermal decomposition of energetic complexes, and the different complexes containing different metals will produce different synergistic effects after mixing.

In 2017, Q. Yang et al. (2017) used the energetic ligand 4,5-ditetrazolylimidazole (H_2_BTI) to synthesize a solvent-free three-dimensional energetic complex [Pb(HBTI)]_n_ by hydrothermal method, the complexes had good thermal stability, high density and insensitivity to friction and impact. The complex can be used as an excellent high-energy-density metal-organic Frameworks material in the field of insensitive burning catalysts. After mixing and burning the complex with AP, it can be seen from the DSC curve that the endothermic peak temperature of the mixed sample is not affected, indicating that the crystal transformation process of AP is hardly affected by the energetic burning rate catalyst. It was worth noting that the two original exothermic peaks of AP merged into a broad peak with a peak temperature of 313°C, which was much lower than the exothermic peak temperature of AP (335°C). Besides, the decomposition heat of AP increased sharply from 1.47 kJ g^−1^–2.62 kJ g^−1^ after the addition of the complex.

**FIGURE 3:**
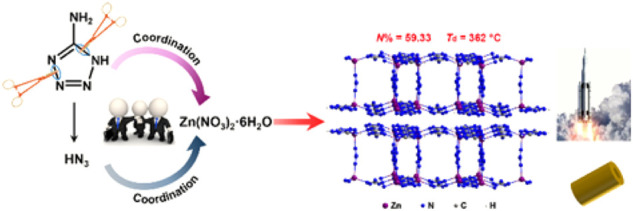
Proposed mechanism for the synthesis of [Zn_2_ (atz)_3_(N_3_)]_n_ (Tan et al., 2022).

In 2022, B. J. Tan et al. (2022) used 5-aminotetrazole (atz) as a ligand to coordinate with zinc ions to obtain a two-dimensional azide-bridged energetic metal-organic frameworks [Zn_2_ (atz)_3_(N_3_)]_n_. This complex can advance the thermal decomposition temperature of AP by about 50°C and the thermal decomposition temperature of hexanitrohexaazaisowurtzitane (CL-20) by about 6°C. In addition, this energetic complex had excellent heat resistance (thermal decomposition temperature was 362°C) and low sensitivity. It was worth mentioning that the authors used only one ligand atz^−^ in the synthesis process, but found two ligands atz^−^ and N_3_^−^ in the complex. The author made a bold speculation on the reaction mechanism through the discovery of two by-products: N_3_^−^ was caused by the partial decomposition of the 5-aminotetrazole ligand during the reaction (Figure 3). Significantly, the author predicted that the complex had excellent detonation performance through Kamlet-Jacob’s equation (Wang et al., 2014), which was comparable to the current detonation velocity of octogen. However, the shortcoming of [Zn_2_ (atz)_3_(N_3_)]_n_ is its low density.

In addition, in 2006, X. Zheng et al. (2006) synthesized 2,4-DNI lead salt (PDNI) from imidazole after nitrification, thermal rearrangement and metathesis. As an energetic burning rate catalyst, PDNI can significantly improve the burning rate of double-base propellants and modified double-base propellants, but the catalytic effect in screw-modified double-base propellant formulations is only comparable to that of inert lead 2,4-hydroxybenzoate. In 2012, Y. L. Wang et al. (Wang Y. et al., 2012) synthesized 4-amino-3,5-dinitropyrazole lead salt using 4-amino-3,5-dinitropyrazole (LLM-116) and Pb(NO_3_)_2_ as raw materials. It was used as a burning rate catalyst in double-base and modified propellants, and its effect on the combustion performance of the two propellants was preliminarily studied. It was worth mentioning that in the pressure range of 6–10 MPa, this burning rate catalyst exhibited “mesa” combustion effect for double-base propellants and a platform combustion effect for modified propellants. The author analyzed that the main reasons for the better catalytic effect of this lead salt burning rate catalyst are as follows: the introduction of energetic groups may accelerate the decomposition of the catalyst during the combustion process, and promote the production of the catalytically active components (lead oxide or lead), which was related to the carbon in the propellant formulation forming a carbon-lead catalytic effect, and effectively regulates the burning rate. In 2021, Y. Zhong et al. (2021) prepared a novel double-ligand energetic complex [Cu(MIM)_2_ (AIM)_2_](DCA)_2_, which contains three kinds of energetic groups (imidazole, nitro and dicyanamide) by reacting 1-methylimidazole, 1-allylimidazole and sodium dicyanamide with copper nitrate. The molecular structure of the complex was determined through single crystal X-ray diffraction analysis, compared it with the single-ligand energetic complex [Cu(AIM)_4_](DCA)_2_ on AP influence law of decomposition temperature, heat release and kinetic parameters in thermal decomposition process. The experimental results showed that the catalytic performance of the double-ligand energetic complex was better than that of the single-ligand energetic complex After adding AP to the double-ligand energetic complex, the exothermic decomposition peak temperature of AP was advanced by 88.8°C, the heat increased by 1676 J g^−1^, and the decomposition activation energy decreased by 47.1 kJ mol^−1^. The reason may be that the different ligands of the dual-ligand energetic complex will interact during the decomposition process, triggering the synergistic effect of the dual-ligand. It can generate more different intermediates and then interact with AP, thereby changing the decomposition process to improve the efficiency of AP decomposition.

Researchers have always pursued the development of high-energy and environment-protecting solid propellants with good combustion performance and less smoke emissions. In recent years, azole-containing metal organic Frameworks have gained popularity due to their high nitrogen and low carbon content, large gas production per unit of mass, high enthalpy and outstanding thermal stability. This kind of organometallic frameworks is expected to be used as a novel energetic burning rate catalyst. Compared with catalysts commonly used for double-base solid propellants (lead salts, copper salts and carbon black), azole-containing metal organic frameworks-based catalysts can provide more energy and have better catalytic performance. However, the application of azole complexes in solid propellants is rarely explored. Therefore, it is necessary to synthesize a series of azole-containing metal organic Frameworks-based energetic complexes and study their catalytic activity. It has important theoretical and practical significance for exploring energetic burning catalysts with promising applications.

#### 2.1.2 Furoxan-containing metal organic frameworks-based burning rate catalysts

The furoxan ring contains 2 C=N bonds and two N–O bonds, and the five atoms are almost located on the same plane. Compared with the high nitrogen compounds of oxazines and azoles, furoxan compounds have higher density and better oxygen balance due to the presence of reactive oxygen species (Sheremetev et al., 2000). However, there are few reports on furoxan and metal coordination compounds on account of the poor ability of furoxan rings to compound with metals.

In 1991, C. Stoner et al. (1991) synthesized four kinds of furoxan compounds [Cu(DAF)_2_(H_2_O)_2_](NO_3_)_2_ [Cu(FP)_2_(CH_3_CN)_2_](NO_3_)_2_ [Cu(FP)_2_(H_2_O)_2_](NO_3_)_2_ and [Cu(FP)_4_(H_2_O)_2_](ClO_4_)_2_CH_3_NO_2_ (DAF, 3,4-diaminofurazan; FP, furazan piperazine). It was found that monomeric furoxan compounds have obvious catalytic effect on solid propellants containing AP, while the synthesized metal furoxan compounds have no obvious catalytic effect.

In 2019, S. Chang et al. (Chang, 2019) synthesized six kinds of Cu(II), Co(II) and Zn(II) complexes using aminofurazan ligands, and tested the catalytic effect of the complexes on AP. All six complexes had excellent catalytic effect on AP, which can advance the high temperature decomposition peak of AP by more than 90°C. Notably, the complex with Cu(II) as the metal center have significantly better catalytic performance for AP than the complexes containing Co(II) or Zn(II). It was noteworthy that [Cu_3_(ZFTO)_2_(OH)_2_·(H_2_O)_2_]_n_ (ZFTO, 5-(1-hydroxytetrazole)-3-aminofurazan) can advance the pyrolysis peak temperature of AP from 442°C to 293.6°C and increase the heat release to 1756 J g^−1^.

In 2021, Y. Li et al. (Li et al., 2021) used 4-amino 3-(tetrazolium) furazan as a ligand to perform a metathesis reaction with copper nitrate in deionized water at 65°C to obtain Cu(NH_3_)_4_ (AFT)_2_ (AFT (4-amino-3-tetrazolium) furazan) complex. The obtained complex had excellent catalytic effect on AP, which can reduce the thermal decomposition temperature of AP by 69°C and increase the heat release to 2711 J g^−1^. The energetic burning rate catalyst can not only reduce the decomposition temperature, but also maintain or even increase the energy of the system. Throughout the thermal decomposition process, the authors observed that the nano-copper oxide underwent changes from columnar, nanotube, and cluster structures. Therefore, the authors speculated that AP reacts with Cu(NH_3_)_4_ (AFT)_2_ during the decomposition process, generating some intermediates with nanofibrous structures, increasing the specific surface area of Cu(AFT)_2_(NH_3_)_4_, thereby improving the adsorption performance of Cu(AFT)_2_(NH_3_)_4_. Finally, the decomposition process of AP was accelareted. This work also provided a new idea for the synthesis of novel energetic burningrate catalysts.

The furoxan ring can improve the density and oxygen balance of MOFs. However, the furoxan-containing MOFs are rarely investigated as a result of the poor ability of furoxan rings to coordinate with metal. Therefore, it is worth exploring how to coordinate furoxan ligands with metal.

### 2.2 Six-membered nitrogen heterocyclic ligand-based burning rate catalysts

#### 2.2.1 Azine-containing metal organic frameworks-based burning rate catalysts

Azines have good stability, high nitrogen content, and high enthalpy of formation. Besides, most azines do not contain nitro groups, so they have low sensitivity. High nitrogen and low hydrogen content in their molecular structure also makes them easy to achieve oxygen balance. However, azine compounds generally have low energy. To tackle this problem, azoles are often introduced into the azine skeleton as organic energy ligands.

**FIGURE 4:**
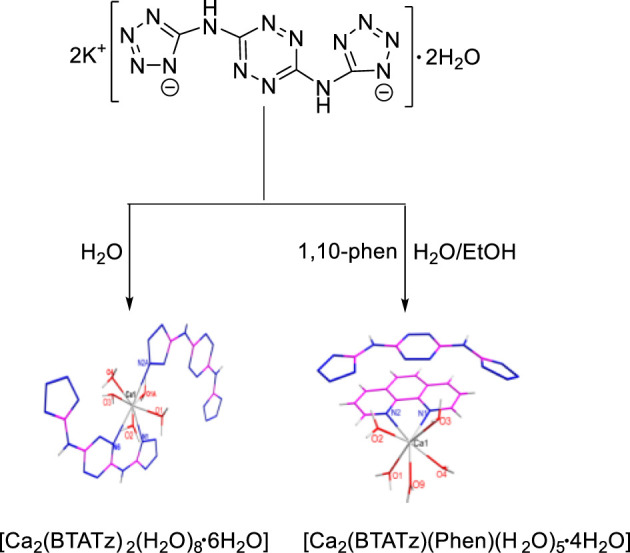
Synthetic route for [Ca_2_(BTATz)_2_(H_2_O)_8_·6H_2_O] and [Ca_2_(BTATz) (Phen) (H_2_O)_5_·4H_2_O] (Liu et al., 2017).

In 2017, F. Q. Zhao et al. (Liu et al., 2017) synthesized two complexes [Ca_2_(BTATz)_2_(H_2_O)_8_·6H_2_O] and [Ca(BTATz) (Phen) (H_2_O)_5_·4H_2_O] based on 3,6-bis(1H-1,2,3,4-tetrazole-5-amino-1,2,4,5-tetrazine (BTATz), and the structures of two complexes were identified by elemental analysis, infrared test and single crystal X-ray diffraction analysis (Figure 4). The single crystal structure showed that the Ca(II) in the two complexes had different coordination configurations [Ca_2_(BTATz)_2_(H_2_O)_8_·6H_2_O] was a symmetrical octahedral coordination configuration, and the coordination atoms included three nitrogen atoms and five oxygen atoms in water molecules. In [Ca(BTATz) (Phen) (H_2_O)_5_·4H_2_O], BTATz existed in the form of anion, while Ca(II) and 1,10-phenanthroline were coordinated with water molecules to form cations. In addition, the thermal safety of the complexes was evaluated by calculating important thermodynamic parameters such as thermal ignition temperature, self-accelerating decomposition temperature and thermal explosion critical temperature. The results showed that the two complexes all had good potential as solid propellants components.

In 2019, S. Wu et al. (2020) synthesized [Co(HTATT)]_n_ using a nitrogen-rich Y-shaped high-energy ligand 3-(5-aminotetrazole)triazine (H_3_TATT) under hydrothermal conditions, which had good thermal stability, low impact sensitivity and friction sensitivity. Compared with the pure AP, AP/[Co(HTATT)]_n_ mixture can combine the two exothermic peaks of AP into a larger peak, and the peak temperature was reduced to 326°C. Moreover, the peak was sharper, and the heat release was increased by 300%. In the same year, S. Wu et al. (2019) also used 3-(5-aminotetrazole)triazine (H_3_TATT) as an energetic ligand to coordinate with Zn(II) and Pb(II) to obtain two energetic complexes [Zn_2_(HTATT)_2_(H_2_O)_2_]3·H_2_O and [PbZn(TATT)_2_(OH) (H_2_O)_n_]. Both of them can accelerate the thermal decomposition of RDX as burning rate catalysts, in which [PbZn(TATT)_2_(OH) (H_2_O)_n_] advanced the decomposition temperature by about 25°C.

Azine ligands in azine-containing metal organic frameworks-based burning rate catalysts usually have high enthalpy of formation, and most of them do not contain nitro groups. Thus, this type of catalyst has low sensitivity and good thermal stability. High nitrogen and low hydrocarbon content in their molecules also makes it easier to achieve high oxygen balance. With high density, positive enthalpy of formation, and good thermal stability, most azine derivatives are considered to be excellent energetic burning rate catalysts. Therefore, the preparation of novel burning rate catalysts from azine derivatives has always been a research hotspot.

#### 2.2.2 Pyridine-containing metal organic frameworks-based burning rate catalysts

Pyridine-containing metal organic frameworks-based burning rate catalysts contains a six-membered aromatic ring, which can generate a large quantity of substances containing carbon when burnt, and promote the catalytic decomposition of the propellant. However, due to the low energy of this ring, adding pyridines to solid propellants can hardly increase the system energy. Therefore, there are few studies on this type of catalyst at present. In order to increase the energy, pyridines are usually nitrated and then coordinated with transition metals. Hydroxypyridine-transition metal complexes are often used as energetic burning rate catalysts. The nitro group contained in these compounds can provide energy, and the hydroxyl group can combine with metal ions.

In 2003, F. Q. Zhao et al. (2003) synthesized six energetic hydroxypyridine lead salts and copper salts, respectively. They selected hydroxypyridine with nitro group as the energetic organic ligand. Herein, the influence of metal lead and copper salts containing energetic pyridine on the combustion catalytic activity of RDX-CMDB was studied. 2-hydroxy-3,5-dinitropyridine lead salts showed excellent ability to regulate the combustion performance of RDX-CMDB propellant, and the pressure index of the solid propellants is only 0.462 at 8–16 MPa.

**FIGURE 5:**
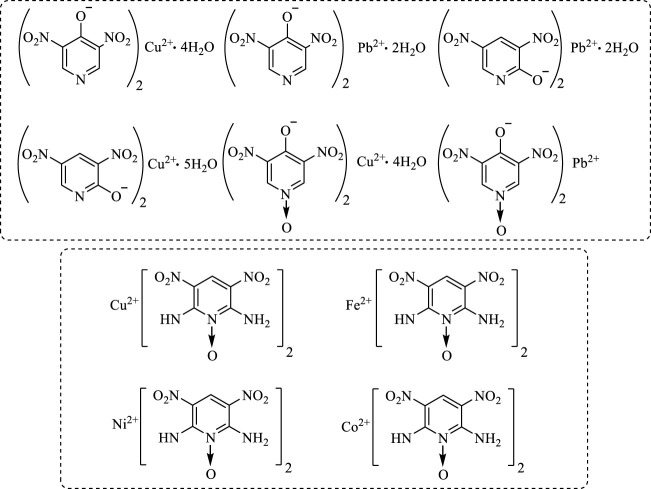
Six energetic hydroxypyridine lead salts, copper salts and ANPyO four energetic metal salts structural formula (Zhao et al., 2003).

In 2012, J. J. Liu et al. (Yang et al., 2013) selected 2,6-diamino-3,5-dinitropyridine-1-oxide (ANPyO) as an energetic ligand to synthesize Cu(ANPyO)_2_, and tested its impact sensitivity, shock wave sensitivity and friction sensitivity. The results showed that the complex was more insensitive than the ligand ANPyO. In addition, the catalytic thermal decomposition of AP by Cu(ANPyO)_2_ complex was studied. The results showed that the complex had a significant promotion effect on the decomposition of AP, which could advance the decomposition peak temperature of AP by 41.33 °C and increase the heat release to 961.35 J g^−1^. In 2015, J. J. Liu et al. (Liu J. et al., 2015; Liu Q. et al., 2016) prepared Ni(II), Cu(II), Co(III) and Fe(III) complexes of 2,6-diamino-3,5-dinitropyridine-1-oxide (ANPyO), and measured the influence law of the four energetic salts on the burning rate and pressure index of the double-base propellant by the target line method (Figure 5). The test data showed that Cu(II) and Fe(III) salts had a significant catalytic effect, which can increase the burning rate of the double-base propellant by 20% and reduce the pressure index significantly at the pressure of 10–20 MPa, showing a good catalytic effect. However, the catalytic effect of Ni(II) and Co(III) salts was not obvious. The author analyzed the reasons for the different catalytic effects of the metal salts on the double-base propellant: the energetic Cu(II) and Fe(III) salts contained energetic groups and released a lot of heat under high temperature, rapidly generating CuO and Fe_2_O_3_ which played a synergistic catalytic role at the molecular level, changed the decomposition process of propellant, and greatly accelerated the thermal decomposition rate of oxidant, thus playing an effective catalytic role.

The excellent catalytic effect of the pyridine-containing metal organic Frameworks-based burning rate catalyst on solid propellants is attributed to the six-membered aromatic ring in the structure. This ring can produce a great number of substances containing carbon during combustion and promote the catalytic decomposition of propellants. However, adding pyridines to the solid propellant can hardly increase the system energy because of the low energy of the pyridine ring. Therefore, it is recommended that energetic groups should be introduced into the pyridine ring and the ring should be coordinated with metal. In this way, the energy level of solid propellants is elevated while the catalytic performance of the catalysts remains unchanged.

### 2.3 Nitroaromatic type burning rate catalysts

**FIGURE 6:**
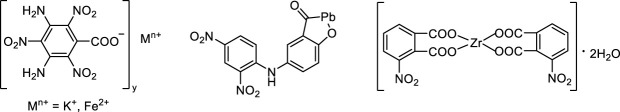
Metal salt of 4-(2,4,6-trinitrophenylamine)benzic acid, zirconium 3-nitrophthalate and 5-(2,4-nitrophenylamino)-lead salicylate (Kulkarni et al., 2005; Song et al., 2007; Zhang et al., 2009).

The application of nitroaromatic energetic metal salts as energetic burning rate catalysts has aroused the interest of researchers. In 2001, J. K. Nair and S. M. Pundlik et al. (Nair et al., 2001; Pundlik et al., 2001) synthesized lead, iron, nickel, copper and cobalt salts of 4-(2,4,6-trinitrophenylamine) benzoic acid (TABA) and several polynitrobenzene energetic burning rate catalysts such as 2,4-nitrogen trinitrophenylamine lead acetate. The stability test results showed that the impact sensitivity of Co^2+^, Fe^2+^ and Ni^2+^ salts of TABA was more than 40 J, and the friction sensitivity was greater than 360 N. They are not sensitive to collision and friction, and can be used safely. Then the physicochemical properties of these energetic catalysts and their catalytic performances in double-base propellants were studied. It was found that these substances can significantly improve the burning rate of the propellants, and the pressure index also declined obviously. The lead salt has the best catalytic effect, which can increase the burning rate of the propellant by 50%–60% in the low pressure range. In 2005, Kulkarni et al. (2005) used 4-(2,4,6-trinitrophenylamine) benzoic acid as an energetic coordination ligand to prepare K^+^ and Fe^2+^ energetic metal salts, and studied the combustion catalytic characteristics of these two energetic salts in AP-HTPB composite propellant. Notably, the experimental results showed that both energetic salts increased the burning rate of AP-HTPB composite propellant by more than 80%, and the pressure index decreased to less than 0.18. In 2007, X. D. (Song et al., 2007) studied an energetic catalyst, benzoic acid 5-(2,4-nitroanilino)-lead salicylate (DNAs-Pb), which can significantly improve the burning of double-base propellant. The catalyst can make the propellant produce the plateau combustion effect in the range of 6–10 MPa and “mesa” combustion effect in the range of 10–14 MPa. In addition, in 2017, Y. L. Wang et al. (2017) continued to synthesize a new type of energetic burning rate catalyst, 1,8-dihydroxy-4,5-dinitroanthraquinone nickel salt by a similar method, and studied its thermal behavior by differential scanning calorimetry at different heating rates. They found that this energetic burning rate catalyst has good thermal stability. In 2009, H. Zhang et al. (2009) prepared zirconium 3-nitrophthalate from 3-nitrophthalate and zirconium nitrate. The structure of zirconium 3-nitrophthalate was characterized by elemental analysis, X-ray fluorescence diffraction, FT-IR and TG-DTG. Then they studied the effect of zirconium 3-nitrophthalate on the combustion performance of double-base and RDX-CMDB propellants. The results showed that zirconium 3-nitrophthalate had a better catalytic effect on the combustion of dual-base propellant, which can significantly increase the burning rate in the low pressure stage and decrease the burning rate pressure index in the middle and high pressure stage. When combined with copper salt, the “synergistic effect” similar to lead salt and copper salt can be produced, which can significantly increase the burning rate in low pressure section and reduce the burning rate pressure index in middle and high pressure section (Figure 6).

Nitroaromatic type burning rate catalysts have become the focus of attention in the field of energetic burning rate catalysts in recent years. This type of catalyst has good stability and compatibility, but low energy due to high carbon content in aromatic rings.

### 2.4 Straight chain nitrogen-rich energetic burning rate catalysts

**FIGURE 7:**
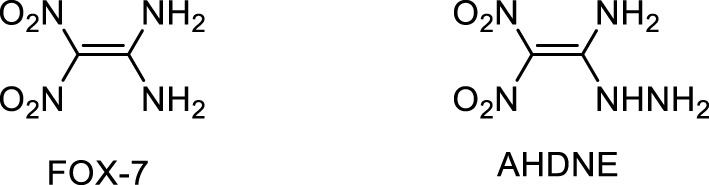
The structures of FOX-7 and AHDNE (Laypov et al., 1998; Ballamy et al., 2002).

Nitrogen-rich straight chain energetic complexes are newly-developed compounds, whose application as burning rate catalysts is still in the early stage. The catalysts are mainly metal salts of 1, 1-diamino-2,2-dinitroethylene (FOX-7) and 1-amino-1-hydrazine-2,2-dinitroethylene (AHDNE) (Figure 7). FOX-7 is a new type of high energy and low sensitivity explosive with superior general properties. It is expected to be one of the main candidate and components of insensitive munitions in the future. Since the first synthesis in 1998, FOX-7 and its metal salts have attracted great attention as an energetic material and burning rate catalysts (Laypov et al., 1998). It is worth mentioning that AHDNE is a derivative of FOX-7. After FOX-7 reacts with hydrazine hydrate in 100°C oil bath for 1 hour, one of the amino groups in the FOX-7 molecule is replaced by the hydrazine group to form AHDNE with o-amino groups. AHDNE is similar to FOX-7 in structure and properties, and both of them belong to the “push-pull” chain alkene (Bellamy et al., 2022).

In 2009, in order to further explore the superior performance of FOX-7 complexes, C. Y. Chang et al. (2008) synthesized six FOX-7 complexes and derivatives, and systematically studied these six complexes: Synthesis, crystal structure, quantitative calculation, thermal decomposition behavior, non-isothermal thermal decomposition kinetics and adiabatic to explosion time of the guanidine salt of FOX-7 [G (FOX-7)], the potassium salt of FOX-7 [K(FOX-7)]H_2_O, 1-amino-1-hydrazine-2,2-dinitroethylene (AHDNE), 1-amino-1-methylamino-2,2-dinitroethylene (AMFOX-7), 2-(dinitromethyl-1,3 diazopentane (DNDZ), 2-(dinitromethyl-1, 3-diazopentane potassium salt [K (DNDZ)]. It was found that the potassium salt of FOX-7 [K(FOX-7)H_2_O] and the potassium salt of 2-(dinitromethyl)-1, 3-diazopentane [K (DNDZ)] had certain flame extinguishing effect. In 2011, X. L. Ren et al. (Ren, 2011)synthesized eight FOX-7 derivatives and their complexes: 1-amino-1-ethylamino-2, 2-dinitroethylene (AEFOX-7), 1-amino-1-(2,4-dinitrophenyl hydrazyl-2,2-dinitroethylene (APHDNE), potassium salt of 1-amino-1-hydrazyl-2, 2-dinitroethylene (AHDNE) [K (AHDNE)], Cesium salt [Cs(AHDNE)], guanidine salt [G (AHDNE)], Rubidium salt [Rb (DNDZ)] of 2-(dinitromethyl-1, 3-diazopentane (DNDZ), cesium salt [Cs(DNDZ)], guanidine salt [G (DNDZ)]. The synthesis, quantization calculation, crystal structure, thermal decomposition behavior, non-isothermal thermal decomposition kinetics and adiabatic to explosion time of the eight compounds were studied. These data provided theoretical basis and performance parameters for the future application of catalysts containing burning rate.

In 2008, C. R. Chang et al. (Chang, 2009) synthesized 1-amino-1-hydrazyl-2, 2-dinitroethylene (AHDNE) from 1,1-diamino-2,2-dinitroethylene (FOX-7), water and hydrazine in aqueous system, and grew single crystal in methanol solution. At the same time, the Gaussian 03 program is applied to the base set level of 6–311 + G(d), and the geometrical optimization of the compound was carried out by HF, MP2 and B3LYP methods. The bond formation and NBO of the compound were analyzed. The molecular structure study showed that 1-amino-1-hydrazine-2,2-dinitroethylene (AHDNE) like FOX-7 was a push-pull nitroamine structure. In 2012, L. Gao et al. (2012) used 1-amino-1-hydrazine-2,2-dinitroethylene (AHDNE) to react with potassium hydroxide or carbonation to obtain the corresponding alkali metal salts AHDNE-K and AHDNE-Cs. Its structure was characterized by elemental analysis and infrared spectroscopy. The effect of two metal salts on the thermal decomposition of HMX, RDX and NC/NG of three modified double base propellants was studied by DSC. The experimental results showed that it has obvious catalytic effect on NC/NG, which reduced the decomposition temperature by about 24°C and increased the energy by 1316 J g^−1^. The copper and lead salts of AHDNE can be used as combustion catalysts, and Cs(AHDNE) may be used as smoke extinguishing agent in the future. Therefore, the study of energetic complexes of AHDNE has great application value.

In summary, the application of NTO-containing EMOFs as energetic burning rate catalysts has been extensively studied. In particular, NTO lead salts have shown excellent catalytic effects on solid propellants. Tetrazole and pyridine-containing EMOFs burning rate catalysts have good stability and compatibility, but there are few studies on the mechanism underlying their catalytic effects on solid propellants. The research of other EMOFs-based burning rate catalysts is scattered. At present, there are still some problems to solve in EMOFs-based burning rate catalysts. First, most of the reported EMOFs-based burning rate catalysts contain lead ions, which are harmful to the environment and health of operators. Besides, lead ions produce white or light blue smoke when combusted, which is not conducive to the stealth and guidance of the missile. Second, most of the energy-containing precursors selected contain moderate or low energy. Third, some ionic salts have no single crystal structure, and their specific composition is unclear.

In the future, researchers of EMOFs-based burning rate catalysts should put emphasis on the following two aspects. On the one hand, to meet the high cleanness, ecological safety, environmental friendliness, and low signal requirements of propellants, it is necessary to seek high energy and insensitive acidic parents and prepare corresponding non-lead green combustion catalysts. It is also important to strengthen the application of the catalysts in propellants. On the other hand, cultivating single crystals to clarify the structure of EMOFs-based burning rate catalysts can make it easier to analyze the mechanism of interaction with metal. Although EMOFs-based burning rate catalysts exhibit excellent performance in catalyzing thermal decomposition of propellant components, most of these catalysts only contain one metal element, consequently exhibiting single catalytic effect. Herein, the catalysts are not applicable to different types of propellants and the practical application is limited.

## 3 Bimetallic multifunctional energetic burning rate catalysts

Bimetallic multifunctional energetic burning rate catalysts are newly developed catalysts, which are rarely studied at present. After the catalyst is combusted, there are two kinds of metal compounds generated, which play a synergistic catalytic effect on propellants. Compared with monometallic compounds, bimetallic complexes have stronger catalytic effects and can better improve the catalytic efficiency of the catalyst. This type of catalyst can broaden the burning range of propellants, reduce the pressure index and improve propellant performance. To promote the application of bimetallic multifunctional energetic burning rate catalysts in solid propellants, future studies should focus on the bimetallic catalysis mechanism and the development of such catalysts.

In 1995, S. W. Li et al. (Li, 1986) found that found that by introducing inert lead salt into the copper-salt-carbon composite catalyst, the lead-copper-salt-carbon ternary composite catalyst could regulate the burning rate of screw pressure RDX-CMDB propellant in a certain range of 3–20 MPa within the range of 5–30 mm/s, and the pressure index can be reduced to less than 0.2. However, it was difficult to reduce the pressure index of the screw-formed Al-RDX-CMDB propellant containing aluminum powder. In order to solve this problem, in 1997, W. W. Li et al. (1997) introduced the energetic lead salt catalyst into the lead-copper-salt-carbon ternary composite catalyst, and a violent “excessive combustion” phenomenon appeared at 4 MPa, herein, the pressure index in the pressure range of 10–18 MPa decreased from 0.46 to 0.36. In addition, the author also compared energetic lead salt catalysts with inert aromatic lead salts, energetic lead salts have better catalytic performance, and they are added in less amount in the formula and have better catalytic performance, and had the outstanding advantage of high burning catalytic activity.

In 2001, S. E. Liu et al. (2001) studied the application of energetic catalysts in high energy and low characteristic signal solid propellants, among which the lead-copper composite salt (NPC) of NTO had the best catalytic effect, and the amount added in the formula can be low to the extent that ordinary inert catalysts are incomparable. The results showed that this energetic catalyst was not only beneficial to improve the formulation energy, but also had a higher catalytic activity, which played a key role in the development of high-energy and low-signal solid propellants.

In 2003, F. Q. Zhao et al. (Chen et al., 2004) found that 4-hydroxy-3,5-dinitropyridine lead salt had relatively good combustion catalytic characteristics for the composite solid propellants formulation containing RDX, but the pressure index was still high at 8–16 MPa. In order to further promote the application process of this burning rate catalyst, according to the Pb-Cu-C synergistic catalysis theory (Li et al., 1995), while 4-hydroxy-3,5-dinitropyridine lead salt was used as the main catalyst, 4-hydroxy-3,5-dinitropyridine copper salt and phthalate copper salt were respectively introduced to form a bimetallic composite catalytic system. The experimental results showed that the composite burning rate catalyst had higher catalytic efficiency for the composite propellant formulation in the measured pressure range. This study also showed that the composite burning rate catalyst system has a more excellent catalytic effect than the single catalyst system (Zhao et al., 2006). In 2007, F. Q. Zhao et al. (2007) studied the influence of energetic bimetallic composite catalysts on the combustion performance of low-smoke propellants, prepared a variety of RDX-CMDB solid propellants containing hydroxy-pyridine lead and copper salts composite energetic catalysts, measured the burning rate of the propellants under different pressures using the target-line method, performed on a linear regression test results. The results showed that the combined use of hydroxypyridine lead salt and copper salt had better catalytic efficiency and lower pressure index than that of single energetic catalyst when the amount of catalyst was constant of the propellants. Furthermore, the authors analyzed the reasons for the excellent catalytic performance of bimetallic energetic burning rate catalysts: the decomposition temperature of copper salts was about 80°C earlier than that of lead salts, and the products of the decomposition of copper salts were copper compounds and carbon, which can prevent the agglomeration of lead compounds and make the catalytic effect of lead compounds play a better role.

In 2011, C. B. Shao et al. (2011) combined 3-nitro-1,2,4-triazol-5-keone lead and copper salt with 4-nitroimidazole lead and copper salts to form a composite catalyst containing energy burning rate. Compared with single-metal catalyst, this catalyst system could further improve the burning rate of the double-base propellants and significantly lower the pressure index of propellant. In addition, the lead/copper/carbon complex system was also studied, which produced an obvious platform effect and reduced the burning residue rate of the double-base propellants to less than 3.0%.

In 2017, S. P. Chen et al. (Zhang Y. et al., 2017) reported bimetallic PbCu(bta)_2_ and [PbCu(bta)_2_(H_2_O)] complexes using bis(1H-tetrazol-5-yl)amine (H_2_bta) as an energetic ligand, and the effects of two bimetallic energetic complexes on the combustion performance of RDX were studied. A small amount of complexes not only advanced the exothermic peak temperature of RDX to 217.5°C, but also increased the exothermic heat of RDX to 1490 J g^−1^. The authors analyzed the reasons for the excellent catalytic properties of this burning rate catalyst: on the one hand, the energetic ligands contained energy groups, which can generate more heat in the combustion process compared with the inert catalysts; on the other hand, the complexes contained two kinds of lead and copper metal ions. The doping of copper ions can reduce the frequent aggregation of lead ions on the surface of RDX during the combustion process, resulting in a synergistic acceleration effect.

**FIGURE 8:**
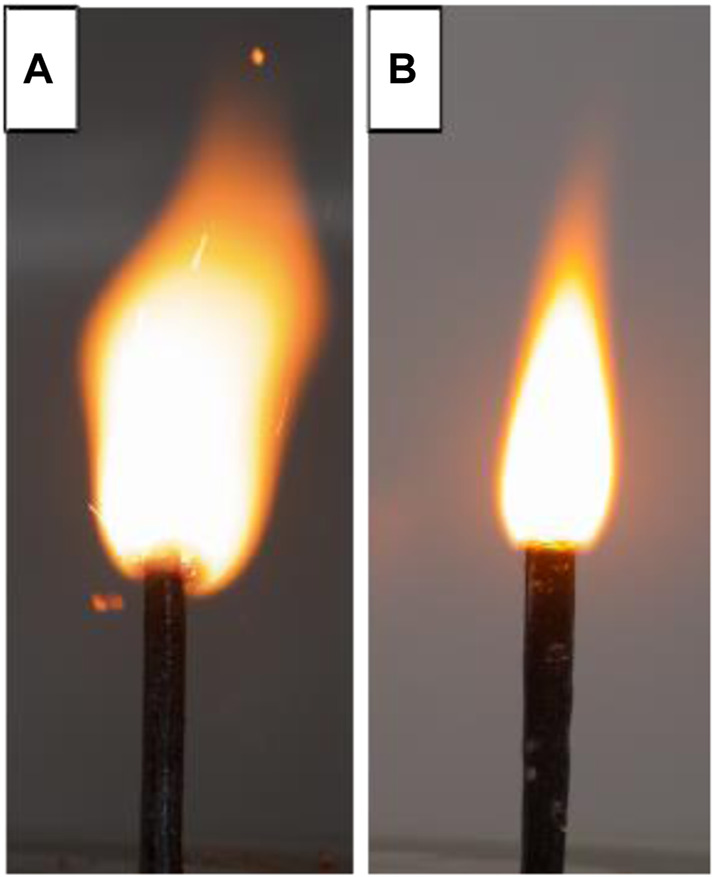
**(A)** Blank propellant; **(B)** Propellant formula containing K_2_Pb [Cu(NO_2_)_6_] (Xia et al., 2020).

In 2014, X. D. Guo et al. (Yang et al., 2014) prepared submicron energetic nitro complexes K_2_Pb [Co(NO_2_)_6_] and K_2_Pb [Ni(NO_2_)_6_] by reversed-phase microemulsion method, and explored the heat of explosion, mechanical sensitivity and catalytic performance of these two energy-containing nitro complexes, as well as their compatibility with HMX, RDX and CL-20. The study of thermal decomposition and adiabatic reaction proved that the two substances can be used as burning catalysts in solid propellants to improve the energy level of the propellant system and have high safety performance. In 2020, X. D. Guo et al. (Xia et al., 2020) used co-precipitation method to prepare K_2_Pb [Cu(NO_2_)_6_] catalyst containing energetic burning rate, and used mechanical ball milling method to refine the complex prepared by co-precipitation method with RDX and HMX were mixed. The thermal decomposition peak temperatures of RDX and HMX were advanced by 34.5°C and 8.1°C, respectively, and the catalytic effect was better. Finally, it was added to HMX/RDX-CMDB propellant, and its static burning rate, flame structure and chemical stability were measured by target line method, single magnification color photography method and methyl violet method. It was worth mentioning that because the added K_2_Pb [Cu(NO_2_)_6_] contains potassium ions, the flame area can be effectively reduced, so as to achieve the effect of flame suppression, therefore, the chemical stability of the propellant is within the permissible range of the standard (Figure 8). In addition, the pressure index of RDX-CMDB propellant can be reduced by 0.111 at medium and high pressure (8–18 MPa).

Ferrocene compounds are a kind of very important commercial burning rate catalysts, but there are obvious sublimation or volatilization phenomena in the process of applying them to propellants, and migration will occur during long-term storage, resulting in propellant combustion instability and affecting the preset ballistic performance of the engine variation (Dilsiz and Ünver, 2010). It was found that ferrocene decomposes into highly active ferric oxide during the burning process, which was more effective than conventional ferric oxide, ferric ferricyanide and ferric ferrocyanide. If transition metal ions are introduced into ferrocene energetic derivatives by coordination, the polarity of ferrocene energetic derivatives will increase after the transition metal ions coordinate with nitrogen and oxygen in ferrocene energetic derivatives. In addition, most of the complexes are non-migration and non-volatile at room temperature, which makes ferrocene energetic complexes become energetic bimetallic burning rate catalysts with high catalytic performance and resistance to migration.

**FIGURE 9:**
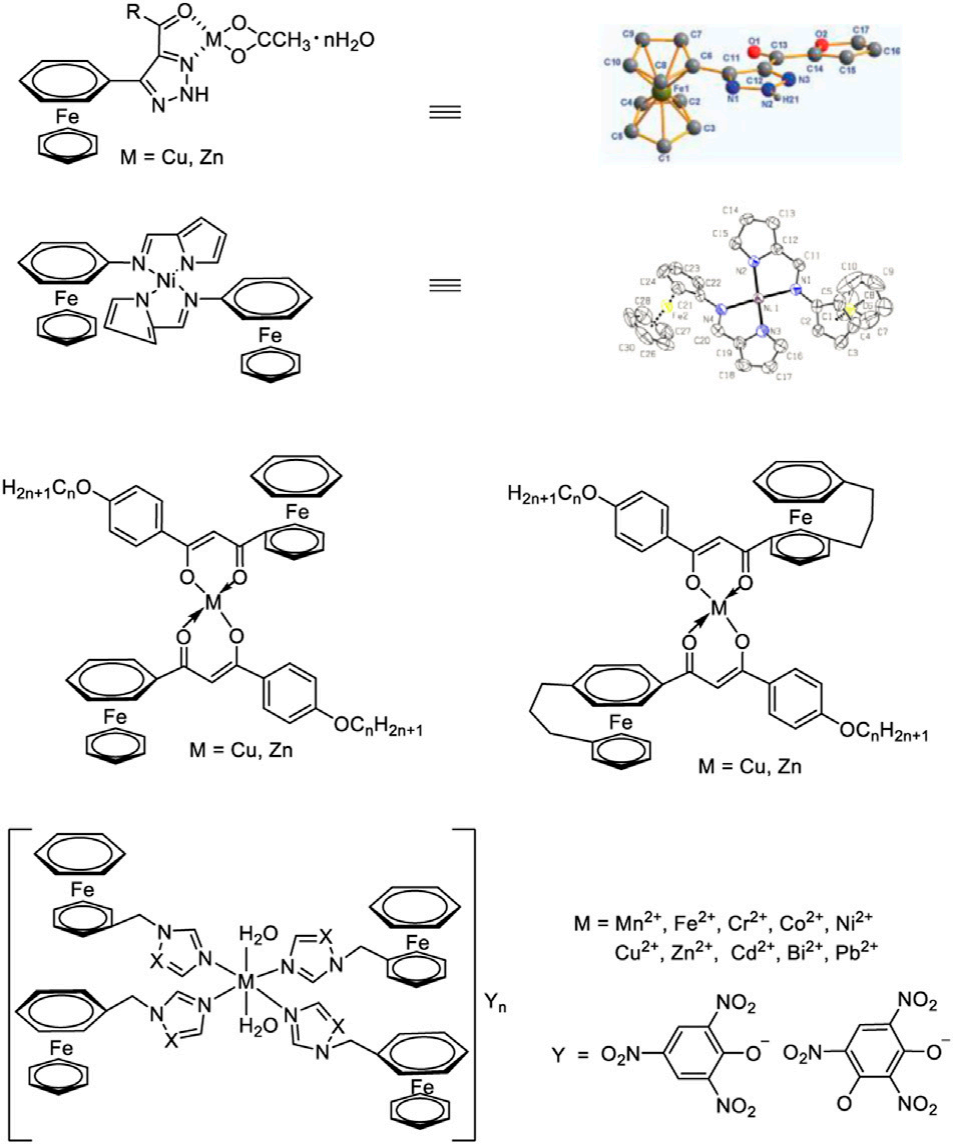
Some ferrocene derivatives (Zhuo et al., 2015; Zhao et al., 2013; Zhao et al., 2015; Jiang, 2019).

In 2011, Y. F. Yuan et al. (Gao et al., 2011; Liao et al., 2012) synthesized three ferrocene dihydropyrazole derivatives with polar groups and two dinuclear ferrocene derivatives (propyl bridged bis-polyferrocene formate) and propyl bridged dimerized ferrocene tetrazole), which advanced the thermal decomposition temperature of AP by more than 50°C and had good catalytic combustion effect on AP. So it was a good catalyst with energetic burning rate. In 2015, Y. F. Yuan et al. (Zhuo et al., 2015)used Schiff base and NiCl_2_.6H_2_O as raw materials to synthesize ferrocene pyrrolimine nickel complexes in moderate yields, and studied its catalytic effect on the thermal decomposition of AP. The results showed that the Ni(II) complex with ferrocenylpyrrole-imine *N*,*N*-chelate exhibited excellent catalytic effect on the thermal decomposition of AP, which can reduce the peak temperature of AP at high temperature by 172°C. In 2013, H. Y. Zhao et al. (Zhao H. et al., 2013) prepared ferrocene-based 1,2,3-triazole compounds and ferrocene-based 1,2,3-triazole Cu(II), Zn(II) energetic complexes, The results show that both types of compounds can effectively reduce the thermal decomposition temperature of AP, among which ferrocene-based 1,2,3-triazole compounds can reduce the decomposition peak temperature of AP by about 30°C, whilethe energetic complexes of ferrocene 1,2,3-triazole Cu(II) and Zn(II) decreased the decomposition peak temperature of AP by 60.6°C and 61.9°C, respectively. The results of this research further show that the bimetallic complex-ferrocene-based 1,2,3-triazole Cu(II), Zn(II) energetic complexes are compared to the single-metal ferrocene-based 1,2,3-triazole compounds have obvious catalytic performance advantages. In 2016, Z. X. Bian et al. (Zhao et al., 2015; Zhao J. et al., 2016) synthesized ferrocenyl *β*-diketone with different substituents and coordinated it with Cu(II), Ni(II) and Zn(II) to obtain a variety of new bimetallic ferrocene complexes. TG-DSC test results showed that the complex effectively reduced the thermal decomposition stability of AP (Cu(II) complex could reduce the peak temperature of AP by 92°C) and release a large amount of heat. In 2018, G. F. Zhang *et al.* (Zhang et al., 2018) used ferrocenyl methyl imidazole and ferrocenyl methyl triazole as energetic ligands to coordinate with Cu(II), Zn(II), Ni(II), Cd(II), Fe(II), Pb(II), Cr(III) and Bi(III), and prepared a variety of bimetallic ferrocenyl energetic derivatives. The effects of these bimetallic complexes on the thermal decomposition process of common oxidants were evaluated. The burning rate catalyst of HTPB/AP propellant system was about twice higher than that of catoxine as burning rate catalyst, which has better application prospect. In 2019, L. P. Jiang et al. (Jiang, 2019) introduced the concept of high nitrogen energetic and bimetallic synergistic catalysis into the design of ferrocene complexes, and designed and synthesized a series of 1,2,4-triazole energetic-ionized ferrocene derivatives. The weight loss rate of thermogravimetric test at constant temperature shows that the weight loss rate of the new complexes was lower than that of catoxine under the same conditions, indi cating that the anti-volatile performance of the complex was better than that of catoxine. The migration resistance was significantly better than ferrocene and catoxine. It was worth noting that all the newly synthesized ferrocene compounds had obvious effects on the thermal decomposition process of AP. And most of the new compounds had better catalytic effect than catoxine. In addition [Cu(FcITz)_2_(H_2_O)_2_](TCP)_2_·H_2_O and [Zn(FcITz)_2_(H_2_O)_2_](ClO_4_)_2_·2H_2_O (FcITz, 3-(ferrocenylmethyl)imino-1,2,4-triazole) (FcATz, 3-(ferrocenylmethyl)amino-1,2,4-triazole) had obvious catalytic effect on HMX, which is relatively rare among the reported ferrocene compounds (Figure 9).

Previous research on bimetallic multifunctional energetic burning rate catalysts concentrated on bimetallic structures, such as lead-copper and iron-copper. Research progress of green inorganic and inert bimetallic combustion catalysts containing bismuth has been made recently. Therefore, future studies should attach importance to the design and synthesis of environmental-protection bimetallic multifunctional energetic catalysts, the exploration of the co-catalytic effect of bimetallic structures, and the discussion of the action mechanism. In addition, the influence of the structure, morphology, particle size distribution and content of the bimetallic multifunctional burning rate catalysts on their catalytic performance should also be investigated in the future (Hou et al., 2021).

EMOFs-based burning rate catalysts are usually energetic salts or complexes prepared by introducing energetic groups (such as ≡N-NO_2_, -O-NO_2_, -N_3_, -N=N-, ≡C-NO_2_, *etc.*) into MOF catalyst molecules. On the one hand, these EMOFs-based burning rate catalysts have high nitrogen content, low carbon and hydrogen content, easy to achieve oxygen balance, and higher gas production per unit mass than ordinary substances. On the other hand, due to the high density of such burning rate catalysts and the large number of -N=N- and C-N bonds with high enthalpy of formation, the catalyst has high enthalpy of formation and thus high energy. Therefore, it can not only adjust the combustion performance of the propellant, but also improve the energy level of the propellant. In other words, EMOFs-based burning rate catalysts have the dual advantages of both high energy and nanometerization, which is a kind of burning rate catalyst with great application potential and needs to be vigorously developed. Its catalytic principle is the same as that of the organometallic catalyst, and the catalytic substance is still the corresponding nanometer or micron grade metal oxide or metal composite oxide decomposed *in situ*. Therefore, it has potential advantages in the field of solid propellants application (Jiang, 2002; Huang et al., 2009; Huang and Zhou, 2012; Zhao et al., 2013a; Chalghoum et al., 2022).

**FIGURE 10:**
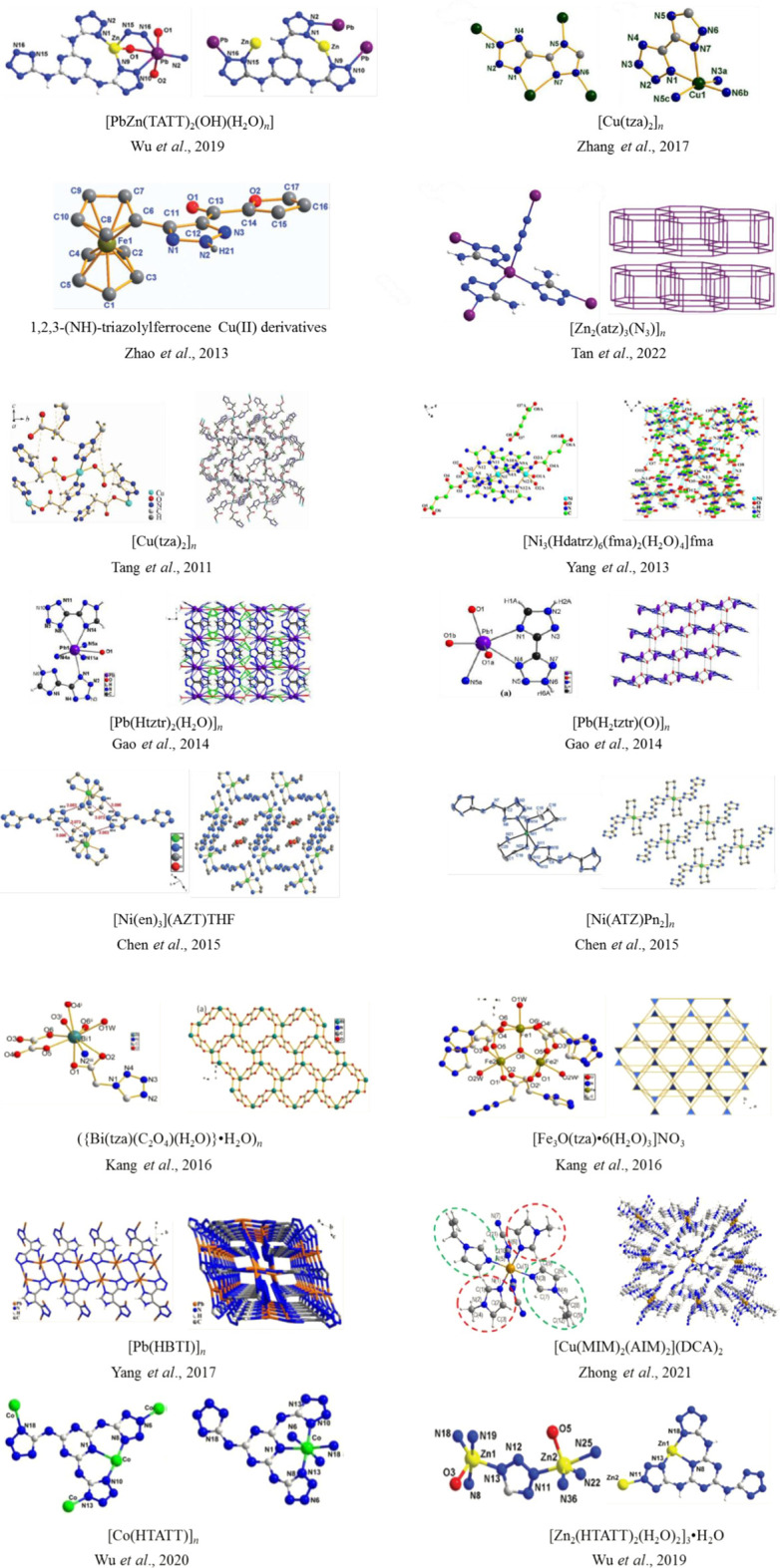
The typical EMOFs structural formula.

So EMOFs can serve as both the burning rate catalyst and energetic additive of solid propellants (Table 2). With various coordination modes, strong structural adjustment ability, and high thermal stability, EMOFs have gradually become an effective way to construct new energetic burning rate catalysts. EMOFs have attracted extensive attention and provide a new approach to synthesizing energetic burning rate catalysts (Figure 10).

**TABLE 2:** The summary of novel energetic burning rate catalysts and some classical energetic materials.

EMOFs	Heating rate/(°C min^−1^)	Catalyst content (mass%)	Apparent activation energy (kJ·mol^−1^)	Preparation procedur	Major advantages	Limits	Ref
{[Cu(tztr)]·H_2_O}_*n*_	10	5	-	Hydrothermal synthesis method	Insensitivity	Containing crystal water	Liu et al. (2015a)
{[Cu(tztr)]}_*n*_	10	5	-	Microcalo-rimetry	Solvent-free, insensitivity and superior thermostability	-	Zhang Y. et al. (2017)
[Cu(tza)_2_]_*n*_	10	50	356.1	Hydrothermal synthesis method	Synthesis procedure is simple	Higher sensitivity	Tang et al. (2011)
[Pb(Htztr)_2_(H_2_O)]_*n*_	10	25	196.1	Hydrothermal synthesis method	superior thermostability and insensitivity	-	Gao et al. (2014)
[Pb(H_2_tztr) (O)]_*n*_	10	25	182.0	Hydrothermal synthesis method	superior thermostability and insensitivity	-	Gao et al. (2014)
[Pb(HBTI)]_*n*_	10	25	-	Hydrothermal synthesis method	Solvent-free and lower sensitivity	-	Yang et al. (2017)
[Zn_2_ (atz)_3_(N_3_)]_*n*_	10	5	219.2	-	superior thermostability and insensitivity	Lower density	Tan et al. (2022)
Cu(AT)_4_H_2_O(ClO_4_)_2_	10	7	-	Hydrothermal synthesis method	Good catalytic activity	Easy to thermal decomposition	Li et al. (2014)
[Ni(en)_3_]AZT·THF	10	25	200.0	Solvent diffusion method	Good catalytic activity	Lower density	Chen et al. (2015)
[Ni(AZT) (pn)_2_]_*n*_	10	25	232.6	Solvent diffusion method	Good catalytic activity	Lower density	Chen et al. (2015)
[Co(HTATT)]_*n*_	10	25	-	Hydrothermal synthesis method	superior thermostability and insensitivity	-	Wu et al. (2020)
[Fe_3_O(tza)_6_(H_2_O)_3_]NO_3_	10	25	-	Hydrothermal synthesis method	Good catalytic activity	Containing crystal water	Li et al. (2016)
[Cu(MIM)_2_ (AIM)_2_](DCA)_2_	10	5	-	Hydrothermal synthesis method	Increase in heat release	Lower density	Zhong et al. (2021)
[Cu(AIM)_4_](DCA)_2_	10	5	-	Hydrothermal synthesis method	Solvent-free	Lower density	Zhong et al. (2021)
(Cu(MIM)_4_](DCA)	10	5	-	Hydrothermal synthesis method	Solvent-free	Lower density	Zhong et al. (2021)
{[Zn_2_(HTATT)_2_(H_2_O)_2_]_3_·H_2_O	10	20	-	Hydrothermal synthesis method	Good detonation velocity and detonation pressure	Containing crystal water	Wu et al. (2019)
[Ca_2_(BTATz)_2_(H_2_O)_8_·6H_2_O]]	10	5	287.2	Mixed solvent evaporation method	Superior thermostability	Containing crystal water	Liu et al. (2017)
[Ca_2_(BTATz) (Phen) (H_2_O)_5_]	10	5	293.6	Mixed solvent evaporation method	Superior thermostability	Lower density	Liu et al. (2017)
[PbZn(TATT)_2_(OH) (H_2_O)_*n*_]	10	20	-	Hydrothermal synthesis method	Good detonation velocity and detonation pressure	Containing crystal water	Wu et al. (2019)
K_2_Pb [Co(NO_2_)_6_]	5	5	-	Mixed solvent evaporation method	Three metal complex catalysis and insensitivity	-	Yang et al. (2014)
K_2_Pb [Cu(NO_2_)_6_]	5	1.15	-	Mixed solvent evaporation method	Composite catalyst with flame extinguishing function	-	Xia et al. (2020)
1,2,3-(NH)-triazolylferrocene Cu(II) complex	10	5	-	Substitution and coordination reactions	Good catalytic activity	Synthesis procedure is tedious	Zhao et al., 2013a
1,2,3-(NH)-triazolylferrocene Zn(II) complex	10	5	-	Substitution and coordination reactions	Good catalytic activity	Synthesis procedure is tedious	Zhao et al., 2013b
FcMTz metal compex	10	5	-	Mixed solvent evaporation method	Synthesis method is simple	Containing crystal water	Zhang J. et al. (2017)
FcMIz metal compex	10	5	-	Mixed solvent evaporation method	Synthesis method is simple	Containing crystal water	Zhang Y. et al. (2017)
NGO/RDX nano-energetic composite	10	2	-	Graphene loading	Excellent catalytic property	Reduce energy level	Yuan et al. (2020)
GO-Cu(II)-AmTZ	10	25	-	Graphene loading	Excellent catalytic property	Reduce energy level	Yang et al. (2020)
Pb(NTO)_2_	10	5	-	Hydrothermal synthesis method	Good catalytic activity	High sensitivity	Huang et al. (2013)
Cu(NTO)_2_	10	5	119.0	Room temperature evaporation process	Ligand is easy to get	Pressure sensitivity	Zhang et al. (1993)
Co(NTO)_2_	10	5	127.5	Room temperature evaporation process	Ligand is easy to get	-	Song et al. (1996)
Ba(NTO)_2_	10	5	-	Room temperature evaporation process	Ligand is easy to get	-	Guan et al. (1999)
2HDNPPB	10	3	246.1	Mixed solvent evaporation method	Add less	Lower energy	Chen et al. (2004)
4HDNPPB	10	3	187.2	Mixed solvent evaporation method	Add less	Lower energy	Chen et al. (2004)
[Ni_3_(Hdatrz)_6_ (fma)_2_(H_2_O)_4_] fma	10	25	96.5	Hydrothermal synthesis method	Good catalytic activity	Higher sensitivity	Yang et al. (2013)
PbTMT	10	2	-	Hydrothermal synthesis method	Good catalytic activity	Ligand synthesis procedure is tedious	Zhao et al. (2004)
CuTMT	10	2	-	Hydrothermal synthesis method	Good catalytic activity	Ligand synthesis procedure is tedious	Zhao et al. (2004)
SrTMT	10	2	-	Hydrothermal synthesis method	Good catalytic activity	Ligand synthesis procedure is tedious	Zhao et al. (2004)
PbPHT	10	2	-	Hydrothermal synthesis method	Good catalytic activity	Ligand synthesis procedure is tedious	Zhao et al. (2004)
PbTABA	10	4	-	Room temperature evaporation process	Ligand is easy to get	Higher sensitivity	Pundlik et al. (2001)
CuTABA	10	4	-	Room temperature evaporation process	Ligand is easy to get	-	Pundlik et al. (2001)
CoTABA	10	4	-	Room temperature evaporation process	Ligand is easy to get	-	Pundlik et al. (2001)
DNAS-Pb	10	3	-	Hydrothermal synthesis method	Higher lead	Lower energy	Song et al. (2007)
HDNPPb	10	25	-	Hydrothermal synthesis method	Ligand is easy to get	Lower energy	Zhao et al. (2003)
HDNPCu	10	25	-	Hydrothermal synthesis method	Ligand is easy to get	Lower energy	Zhao et al. (2003)
HDNPOPb	10	25	-	Hydrothermal synthesis method	Ligand is easy to get	Lower energy	Zhao et al. (2003)
HDNPOCu	10	25	-	Hydrothermal synthesis method	Ligand is easy to get	Lower energy	Zhao et al. (2003)
ANPyOCu	10	2	187.1	Hydrothermal synthesis method	Good catalytic activity	Lower energy	Liu et al., 2015b
ANPyONi	10	2	-	Hydrothermal synthesis method	-	Poor catalytic effect	Liu et al., 2015a
ANPyOCo	10	2	-	Hydrothermal synthesis method	-	Poor catalytic effect	Liu J. et al. (2016)
PDNI	10	1.3	-	Hydrothermal synthesis method	Lower sensitivity	Lower energy	Zheng et al. (2006)
PDNAP	10	2.5	192.83	Hydrothermal synthesis method	High yield	-	Wang C. et al. (2012)
DAP-4	10	2.5	-	Hydrothermal synthesis method	High yield and excellent catalytic property	A bit higher sensitivity	Chen et al., (2018a)

## 4 Carbon-supported EMOFs burning rate catalysts

Because carbon-based materials have a very large specific surface area, the exhibit both adsprption and catalytic properties, so it can be used as an inert burning rate catalyst, but the catalytic effect was not good. Therefore, carbon materials are often used in conjunction with EMOFs for better catalytic effect. The action mechanism of carbon materials and EMOFs is as follows: carbon layer is a catalytic bed enriched in metal lead and copper, which can block metal condensation; Moreover, it can inhibit the escape of aldehyde, NO, NO_2_ and other gases, so that it can fully react in the condensed phase, with co-catalytic effect; At the same time, carbon is a highly effective reducing agent of NO, NO_2_ and PbO. Therefore, the carbon-based supported burning rate catalysts developed in recent years have very excellent catalytic effects in the field of solid propellant catalysis, especially graphene oxide, which has attracted great attention in the field of solid propellants (Zhao et al., 2000; Zhao et al., 2013b; Liu, 2021; Zhang et al., 2021; Muller and Gany*,* 2022).

Graphene oxide (GO) has a stable structure, a large specific surface area, high thermal and electrical conductivity. The layered molecule structure of GO contains such functional groups as carboxyl and hydroxyl groups, which enable GO to combine with a variety of energetic groups through covalent bonds (Yan et al., 2016a). GO has been widely used for desensitization of energetic materials and catalytic reactions. GO-supported energetic burning catalysts have excellent burning catalytic effects, high structural thermal stability, and multiple modification effects on solid propellants (Mccrary et al., 2012; Li Y. et al., 2015; Yan et al., 2016b).

**FIGURE 11:**
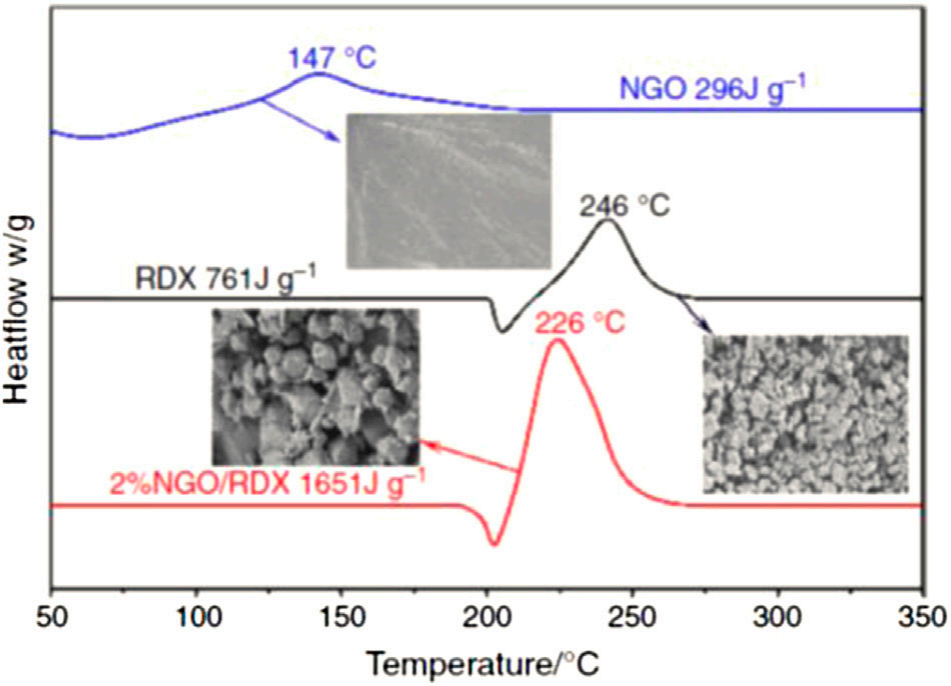
Dsc of NGO, RDX and NGO/RDX (Yuan et al., 2020).

In 2020, S. Yuan et al. (2020) synthesized nano nitrified graphene oxide (NGO) by nitrifying graphene oxide (GO) with nitrified sulfur mixed acid, and introduced it into RDX as a new nano energetic combustion catalyst by solven-antisolvent method to obtain NGO/RDX, and confirmed that monolayer graphene was coated on RDX by scanning electron microscopy. It was found that the introduction of 2% NGO into RDX could achieve a satisfactory catalytic effect and significantly improve the thermal decomposition efficiency. The decomposition peak temperature of pure RDX decreased from 246°C to 224°C, and the heat release increased from 761 J g^−1^–1651 J g^−1^ in 2% NGO/RDX composite (Figure 11). In addition, the introduction of 2% NGO also significantly reduces the impact sensitivity and friction sensitivity of RDX, which was an excellent energetical burning rate catalysts.

In 2020, S. P. Chen et al. (Yang et al., 2020) used graphene oxide (GO) to be complexed with Cu(II) ions in copper nitrate hexahydrate, and then combined with the energetic ligand 3-amino-1,2,4-triazole (AmTZ) coordination to obtain a heat-resistant and insensitive ECP composite (GO-Cu(II)-AmTZ). When the heating rate was 10°C/min, its exothermic decomposition peak temperature T_*p*_ was 318.13°C, and the heat of decomposition was as high as 6,805.17 J g^−1^. Moreover, when GO-Cu(II)-AmTZ was mixed with AP as a burning rate catalyst, the low temperature decomposition peak of AP at 335.4 °C disappeared, and the high temperature decomposition peak of 441.3°C was advanced to 298.4°C. In addition, the AP activation energy was reduced from 168.7 kJ mol^−1^–122.4 kJ mol^−1^. These results indicated that the ECP composite (GO-Cu(II)-AmTZ) has great application potential as a burning rate catalyst. In 2021, Q. L. Yan et al. (Wang et al., 2021) prepared three high-energy insensitive graphene-based energetic coordination complexes GO-CHZ-Co, GO-CHZ-Ni and GO-CHZ-Cu (carbohydrazide (CHZ)) according to the literature. The performance of the four-component composite propellant under the action of this kind of catalyst was comprehensively evaluated from the surface morphology, thermal decomposition characteristics, gas-phase decomposition products, burning performance of the polymer, and the preliminary action rule was obtained. All three energetic coordination polymers can increase the burning rate of the four-component composite propellant more obviously and increase the pressure index. Intriguingly, GO-CHZ-Cu made the propellant maintain a relatively low pressure index (0.34) while increasing the overall burning rate of the propellant by 46.4%, increasing its heat release to 3,064.1 J g^−1^. It was expected to be used as a new type of energetic burning rate modifier in butary-hydroxyl quaternary-component propellant. In addition, GO-CHZ-Cu can make the Al powder burn more completely and the combustion efficiency was higher.

S. Hanafi (Hanafi et al., 2020) successfully prepared new highly energetic coordination polymers (ECPs) with THNO_3_-doped metal perchlorate complexes (Zn, Co, Ag, and Fe) by a facile chemical modification method based on functionalized graphene oxide (GO) sheets. During the complexation reaction, crystal clusters of the T-M complex are grafted onto the GO-T surface or between the GO-T layers. The density of the resulting catalyst increases due to molecular packing between the functionalized GO layers. The incorporation of additional THNO_3_ significantly increases the energy content of the new catalysts. The use of transition metal perchlorate instead of nitrate has a significant effect on the decomposition of AP and RDX. The distribution of different catalysts on AP and RDX surfaces is uniform. The use of different transition metal complexes has a great influence on the AP decomposition mechanism. The as-prepared GO-T-Zn-T exhibited a good effect on the thermal decomposition of AP (T_*p*_ = 194°C), while there was a slight difference in the RDX decomposition pathway. In particular, the heat released from the decomposition of AP/GO-T-Zn-T (2495 J/g) was significantly higher than that of pure AP (1076 J g^−1^). S. Hanafi (Hanafi et al., 2022a) continue studied the catalytic activity of the energetic coordination polymer containing graphene oxide (GO-T-Fe-T) (T-Fe) on the high energy eutectic pyrolysis of hydrazine 3-nitro-1,2,4-triazole-5-one and ammonium nitrate (HNTO/AN). Four equal inverse integral dynamics methods wre used to calculate the kinetic triplet. The experimental results showed that both T-Fe and GO-T-Fe-T had good catalytic activity. When T-Fe and GO-T-Fe-T were introduced, the decomposition temperature and activation energy of the cocrystal were decreased by 31 kJ mol^−1^ and 55 kJ mol^−1^, respectively. Compared with T-Fe, GO-T-FE-T greatly reduces its T_*p*_ by increasing its activation energy, thus improving the cocrystal stability, which may be due to the enhanced thermal conductivity and heat transfer capacity of GO.S. Hanafi (Hanafi et al., 2022b) investigated the properties of composite solid-based propellant (CSP) with hydrazine 3-nitro-1, 2, 4-triazo-5-one and ammonium nitrate (HNTO/AN) cocrystal as oxidant, hydroxy-terminated polybutadiene (HTPB) as a binder, with or without nanocatalites (triaminoguanidine-transition iron, containing graphene oxide). Compared with the AP/HTPB baseline formulation, the developed baseline formulation has promising properties as it can provide an interesting specific pulse value (231 s^−1^). On the other hand, based on DSC data, four model-free integration methods, namely it-KAS, it-FWO, VYA, and TAS were used to determine the kinetic triplets of the studied samples. The results showed that the decomposition peak temperature (T_*p*_) and activation energy (E_a_) decreased significantly after the addition of nanocatalyst. This research would certainly drive further research in the field of high-energy co-crystals and graphene-based materials that are expected to be used in solid rocket propellants formulation. It was worth noting that the authors hypothesized that the excellent performance of GO-T-Fe-T nanocatellite for the reduction of E_a_ might be due to the stronger interaction between GO-T and T-Fe. However, additional studies related to the evaluation of combustion and mechanical properties are needed in the future to better understand this type of solid propellant. In addition, Dissimilar to the widely used supported nano-metal burning rate catalysts, the application of carbon-supported EMOFs burning rate catalysts in solid propellants is still at a preliminary stage. The preparation and application of this type of catalyst have become one of the development directions of burning rate catalysts. The computation of the quantity of the supported catalyst and the loading uniformity of GO in the cavity during the loading process of GO has important influence on the application of this type of catalyst. In addition, to promote the application of the catalyst, the preparation cost of GO should be reduced.

## 5 Catalysts that can be compounded with EMOFs

Perovskite composite oxides, borane energetic ionic liquids (salts) and polymer-supported burning rate catalysts have potential application prospects in the field of solid propellants. Perovskite composite oxides compounds have similar structures to EMOFs and good thermal stability. Besides, these compounds are environmentally friendly as they do not contain heavy metals. A series of energetic perovskite composite oxides have been developed in recent years. They can compound with EMOFs to constitute multimetallic synergistic energetic burning rate catalysts. The energetic ionic liquids (salts) of boranes have broad application prospects, especially the carborane derivatives with closed cage structure and ionic hydroborates. When the carborane cage is destroyed, tension energy and binding energy will be released, which promote the enrichment of the catalyst and block the condensation of new ecological metals in the reaction. Energetic ionic liquids (salts) of boranes have relatively high adsorption and catalytic performance. They can promote the combustion of the propellant, induce the Mersa effect, significantly reduce the propellant pressure index, and greatly increase the burning rate of propellants under low pressure. It is predicted that the integration of energetic ionic liquids (salts) of boranes and EMOFs can notably improve the catalytic performance. The grafting of ferrocene burning rate catalysts into prepolymers has the dual effects of adhesion and combustion promoting. It provides a reference for preparing multifunctional composite burning rate catalysts by graft copolymerization of EMOFs and adhesives in the future. This is also in line with the recent development trend of multifunctional composite burning rate catalysts, which are the focus of future research.

### 5.1 Perovskite complex burning rate catalysts

**FIGURE 12:**
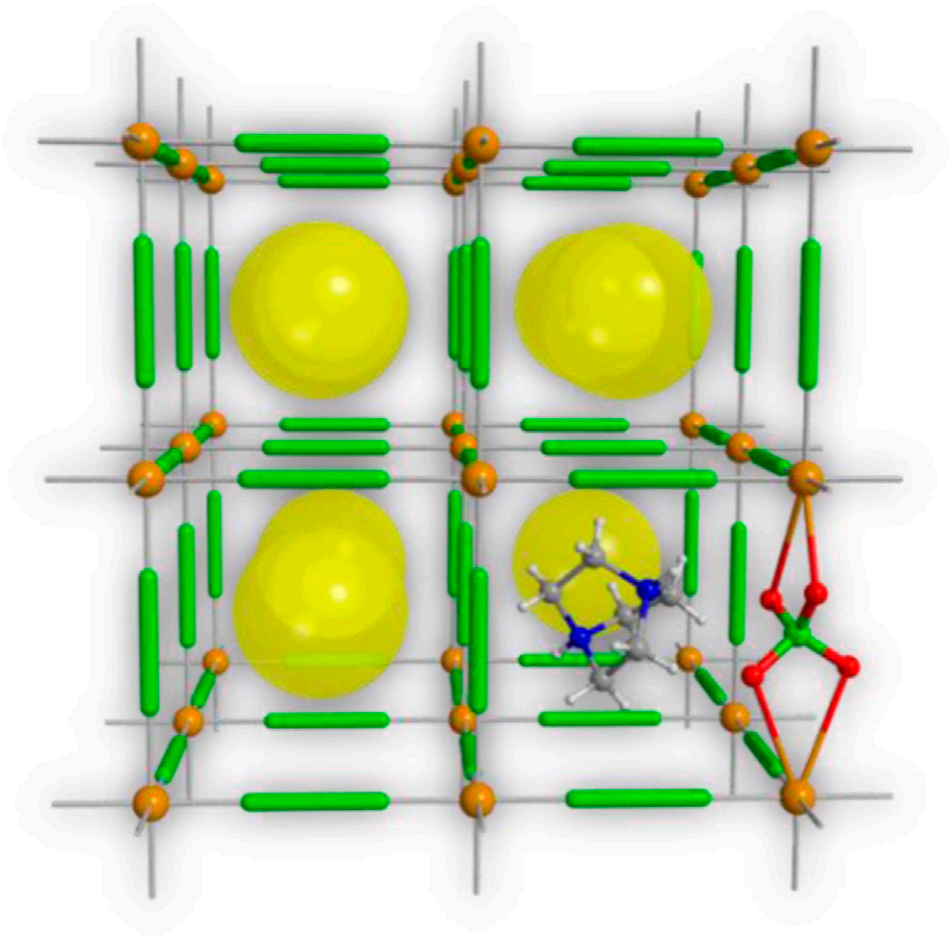
Schematic diagram of molecular perovskite-type energetic material structure (Chen et al., 2018b).

Perovskite complexes (ABO_3_ type) are a novel EMOFs-like material with unique physical and chemical properties. The A site is usually a rare earth ion or an alkaline earth element ion, and the B site is a transition element ion. Both the A and B sites can be partially replaced by other metal ions of similar radius to keep the crystal structure basically unchanged. Therefore, different structures can be derived by substituting A and B sites to further improve the performance of catalyzing the propellant combustion (Zhang W. et al., 2020). In recent years, perovskite-type energetic materials have attracted increasing attention. Moreover, as energetic materials, they also have excellent detonation performance (Chen et al., 2018b; Zhang et al., 2022). Therefore, the use of perovskite-type complexes as burning rate catalysts for solid propellants has aroused great interest, but relevant reports are few (Figure 12).

In 2007, Z. X. Wei et al. (Wei et al., 2007; Wei et al., 2010a) prepared perovskite-type superfine complex LaCoO_3_ by sol-gel method with stearic acid as complexing agent, and preliminaries studied the effect of LaCoO_3_ powder on the combustion performance of nitrosamine modified double-base propellant. The results showed that ultra-fine LaCoO_3_ with a mass fraction of 1.7% can significantly reduce the pressure index and increase the burning rate at 3–18 MPa. Therefore, the platform combustion effect occured at 14–16 MPa. Z. X. Wei et al. (Wei et al., 2009; Wei et al., 2010b; Wang et al., 2010) also synthesized a series of perovskite-type complexes such as LaMnO_3_, LaFeO_3_, La_0.8_Sr_0.2_MnO_3_ and LaOCl by combustion synthesis, and studied their effects on the catalytic thermal decomposition process of RDX and HMX. The results showed that these catalysts can increase the thermal decomposition activity of RDX and HMX and accelerate the combustion rate. It can be seen that perovskite-type complex had good application prospect in the field of solid propellants. In 2008, X. D. Guo et al. (2008) prepared perovskite-type nano NdCoO_3_ with an average particle size of 10 nm and better dispersion by salt-assisted solution combustion method, subsequently, studied the influence on the thermal decomposition of AP and double-base adhesive. The study showed that the catalytic efficiency was the best when the mass fraction was 3%, and with the increase of the mass fraction of nano NdCoO_3_ catalyst, the catalytic efficiency first increased and then decreased. At the same time, the catalytic amount of nano NdCoO_3_ can greatly reduce the high temperature decomposition peak temperature of AP. On the one hand, Nd and Co in nano NdCoO_3_ has the synergistic catalytic effect. On the other hand, nanometer NdCoO_3_ has a large catalytic specific surface area. Notably, author also explored the combustion catalytic effect of nano NdC_O_O_3_ on double-base adhesives bonded by small balls. The combustion rate of high-burning propellant bonded by small balls was higher, and the decomposition peak temperature of double-base adhesives was also decreased. Moreover, the apparent decomposition heat of the two was increased and the pressure index was lower.

In recent years, X. M. Chen’s team has synthesized a series of molecular perovskite-type energetic materials. These energetic materials are a kind of three-dimensional compact crystal structure, which is formed by the crystal self-assembly strategy, and the reducing components are embedded in the cage constructed by the oxidizing components at the molecular scale. This kind of multi-component energetic compound with host and guest structure has the characteristics of no moisture absorption, simple preparation process and economy, and has outstanding performance in thermal stability, showing better comprehensive performance. Recently, X. M. Chen *et al.* conducted a preliminary study on the application of molecular perovskite-type energetic materials in the field of burning rate catalyst. Preliminary experimental results showed that (C_6_H_14_N_2_)NH_4_(ClO_4_)_3_ (DAP-4) in molecular perovskite-type energetic materials had a significant catalytic effect on the burning rate of composite modified double-base propellant. In the pressure range of 1–22 MPa, the burning rate at different pressures was basically increased, especially at high pressure (10–22 MPa), the burning rate was greatly increased (Shang et al., 2022; Shang et al., 2020a; Shang et al., 2020b; Chen et al.,2018a; Chen et al., 2022; Zhang J. et al., 2020).

### 5.2 Borane energetic ionic liquid (salt) burning rate catalysts

Studies suggest that boron-hydrogen burning rate catalysts can effectively increase the burning rate of solid propellants (Zheng and Wang, 1989). There are many types of boron-hydrogen compounds that can adjust the burning rate of solid propellants. Carborane derivatives with closed cage structure and ionic hydroborates are the most extensively studied compounds (Shan et al., 1996; Chen et al., 2000). The new borane ionic liquids (salts) developed in recent years have good application prospects and have been used as high energy rocket propellants. However, related research is still in the infancy.

In 2009, a research report of the United States Air Force Research Laboratory (AFRL) pointed out that ionic liquids can be used as new propellant additives due to their unique properties, and the corresponding properties of synthetic energetic ionic liquids as high energy rocket propellants were characterized (Wang Y. et al., 2012). Due to the adjustable nature of borane energetic ionic liquids, some ideal structures can be designed by selecting suitable anions and cations, so that some ionic liquids (salts) can be prepared as high energetic catalysts and applied to increasing the burning rate of modified propellants. At present, a series of energetic ionic salts have been prepared by combining nitrogen-containing heterocyclic cations with B_10_H_10_^2-^ or B_12_H_12_^2-^ hydrogen borate ions, and their properties as propellants, additives and explosives have been studied. For the preparation of this kind of energetic ionic salt, the methods used are generally double decomposition reaction and acid-base neutralization into salt. In 2009, M. Nieuwenhuyzen *et al.* (Nieuwenhuyzen et al., 2009) prepared a series of low melting point ionic liquids by combining imidazole cations with boron cluster anions through the metadecomposition reaction. The introduced boron cluster anions were [Co(C_2_B_9_H_11_)_2_]^-^ [C_2_B_9_H_12_]^-^, [B_10_Cl_10_]^2-^ [B_12_Cl_12_]^2-^. It was found that the ionic salt with [B_12_Cl_12_]^2-^ had a higher thermal decomposition temperature. What’s more, in 2010, S. A. Shackelford et al. (Shackelford et al., 2009; Shackelford et al., 2010) prepared a series of heterocyclic carborane ionic salts by combining a variety of unsaturated high-energy heterocyclic cations including triazole cations and imidazole cations with [B_12_F_12_]^2-^ [B_12_H_12_]^2-^ and [CB_11_H_12_]^-^ using a new process. This kind of material had the characteristics of high energy, high density, and can be used as burning rate catalyst for high density propellant.

### 5.3 Polymer-supported burning rate catalysts

Ferrocene-based small molecules have demerits of high product mobility, high output of by-products, and the difficulty in purifying and separating products. In order to improve the catalytic performance and reduce the influence of migration on burning rate catalysts, ferrocene polymers have been studied. Although most of the polymer-supported burning rate catalysts reported in literature do not contain energy, these catalysts provide a basis for the development of polymer-supported EMOFs.

**FIGURE 13:**
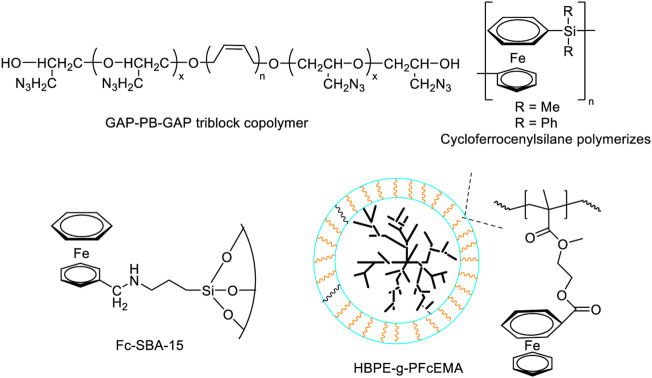
Partically loaded burning rate catalyst (Wasudevan and Sundararajan, 1999; Fu et al., 2008; Jiang, 2002; Deng et al., 2015).

In 1992, Manners et al. (Daniel et al., 1992) synthesized poly (ferrocene dimethylsilane) with molecular weight greater than 10^5^ by thermal initiation ring opening polymerization with silicon dimethyl ferrocene bridge as raw material. The synthesis of this compound opened the beginning of the synthesis, characterization, performance and application of polymeric macromolecular ferrocene burning rate catalyst. In 1999, V. Vasudevan et al. (Vasudevan and Sundararajan, 1999) synthesized GAP-PB-GAP triblock copolymer through cationic ring-opening polymerization with BF_3_·Et_2_O as catalyst, which could be used as energetic binder for HTPB-AP propellant. At the same time, the thermal decomposition data showed that the copolymer added to HTPB-AP propellant can also significantly increase the burning rate of the propellant. Inspired by the above work, in 2003, K. Subramanian et al. (Fu et al., 2008) synthesized a new type of ferrocene burning rate catalyst with ferric tricarbonyl and HTPB as raw materials. However, the application of ferric tricarbonyl in propellants is limited due to its high toxicity and unstable existence under general conditions. In the same year, D. Saravanakumar et al. (Jiang, 2002) connected ferrocene to HTPB and synthesized a non-migrating grafted ferrocene burning rate catalyst (Fc-HTPB). It was found that the viscosity of the polymer will increase due to the increase of its crosslinking degree when the iron content exceeds 2%. Once cured, the substance was insoluble in any solvent. Because iron tricarbonyl was particularly easy to decompose and the thermal stability of the compound was poor, which limited its further application possibility. In 2008, D. Saravanakumar et al. (2010) synthesized a polymer with high iron content and difficult migration. This compound was synthesized by grafting ferrocene onto HTPB through F-C alkylation reaction using Lewis acid AlCl_3_ as catalyst. The compound acted as both a burning rate catalyst and an energetic binder in solid propellants. In addition, In 2015, Z. Deng et al. (2015) synthesized and characterized high molecular weight ferrocene functionalized hyperbranched polyethylene (HBPE-g-PFcEMA). TG test results showed that the polymer had high thermal stability, and it still did not migrate at 210°C. When 17wt% HBPE-g-PFcEMA was added to AP, the final decomposition temperature of AP decreased from 78°C and the maximum weight loss temperature of AP decreased to 80°C. Furthermore, compared with ferrocene, HBPE-g-PFcEMA had lower mobility and no migration phenomenon was observed after being placed at 50°C for 31 days (Figure 13).

Energetic Metal Organic Frameworks (EMOFs) have a large specific surface area, a regular pore structure and highly dispersed active sites on the surface. So EMOFs has unparalleled advantages in the field of burning rate catalyst in solid propellants (Table 3). On the one hand, the large specific surface area enables EMOFs to have more sufficient contact with other components of solid propellant. On the other hand, the regular pore structure can make the particle size distribution of EMOFs uniform, and it is easier to change the decomposition process of solid propellants on the subsurface reaction zone or combustion surface.

**TABLE 3:** The physicochemical properties of novel energetic burning rate catalysts and some classical energetic materials.

EMOFs	*ρ* ^a^/g·cm^−3^	*N* ^b^/(%)	*Ω* ^c^/(%)	*T*_*d*_ ^d^/(°C)	*Q* ^e^/(kJ·g^−1^)	*D* ^f^/(km·s^−1^)	*p* ^g^/(GPa)	*IS* ^h^/(J)	*FS* ^i^/(N)	Ref
{[Cu(tztr)]·H_2_O}_*n*_	2.316	45.23	-48.00	325	5.53	7.92	31.99	>40	>360	Yang, (2019)
[Cu(tza)_2_]_*n*_	2.435	49.08	-56.09	355	16.6	10.40	56.48	32	>360	Zhang J. et al. (2017)
[Pb(Htztr)_2_(H_2_O)]_*n*_	2.159	39.40	-45.03	340	5.69	7.715	31.57	>40	>360	Gao et al. (2014)
[Pb(H_2_tztr) (O)]_*n*_	3.511	27.20	-28.86	318	3.93	8.122	40.12	>40	>360	Gao et al. (2014)
{[Cu_4_(tza)_6_(OH)_2_]·H_2_O}_*n*_	1.892	52.72	-60.24	345	8.91	8.18	30.57	>40	>360	Qu et al. (2017)
[Pb(HBTI)]_*n*_	3.186	34.22	-46.90	325	4.85	7.84	35.87	>40	>360	Yang et al. (2017)
[Zn_2_ (atz)_3_(N_3_)]_*n*_	1.541	59.33	-32.50	362	14.36	8.034	25.96	>40	>360	Tan et al. (2022)
[Co(HTATT)]_*n*_	1.903	65.25	-58.00	374	17.71	9.99	45.71	>40	>360	Wu et al. (2020)
{[Zn_2_(HTATT)_2_(H_2_O)_2_]_3_·H_2_O	1.980	57.48	-43.21	267	8.33	8.69	35.41	>40	>360	Wu et al. (2019)
[PbZn(TATT)_2_(OH) (H_2_O)_*n*_]	2.901	39.72	-29.80	288	4.04	7.84	34.75	>40	>360	Wu et al. (2019)
[Cu(atz) (NO_3_) (OH)]_*n*_	2.41	30.91	-24.72	280	3.16	7.459	29.71	11	180	Su et al. (2017)
[Cu(atrz) (IO_3_)_2_]_*n*_	3.168	19.40	-12.47	267	-	7.271	40.61	18	60	Zhang J. et al. (2020)
[Ag (atrz)_1.5_(NO_3_)]_*n*_	2.160	43.76	-49.99	250	5.78	7.773	29.70	30	-	Yang et al. (2013)
[Ag_2_ (DTPZ)]_*n*_	2.812	32.58	-52.10	346	10.15	8.355	38.39	>40	>360	Qiao et al. (2020)
[Fe (ATZ) (H_2_O)_4_·2H_2_O]	1.807	42.67	-24.39	115	6.99	8.83	34.54	-	-	Liu and Chen, (2018)
[Cs(ABTNA)H_2_O]_*n*_	2.413	34.9	-35.45	225	-	6.780	23.9	>40	>360	Wang et al. (2020)
[Mn_5_ (mtz)_6.1_ (atz)_2.9_(NO_3_)]	1.914	51.05	-57.78	360	5.922	7.551	26.16	>40	>360	Cho et al. (2022)
[Zn (DAF) (H_2_O)_4_](NO_3_)_2_	1.901	13.30	-4.43	235	8.890	8.489	35.33	-	360	Gong et al. (2021)
[Cu(ntz) (N_3_) (DMF)]_*n*_	1.801	38.5	-60.3	279.5	7.57	6.08	16.04	>40	>360	Qu et al. (2016)
[Cu(ntz) (N_3_) (H_2_O)]_*n*_	2.218	41.5	16.9	287.3	0.075	2.22	4.46	>40	>360	Qu et al. (2016)
[Cu(ntz)]_*n*_	2.428	31.8	-22.6	315	6.15	7.97	33.10	>40	>360	Qu et al. (2016)
ZnHHP	2.117	23.61	-49.99	293	2.93	7.016	23.58	-	-	Bushuyev et al. (2013)
CHP	1.948	14.71	-11.48	194	5.23	8.225	31.73	0.5	-	Bushuyev et al. (2012)
CHHP	2.000	23.58	-13.05	231	3.14	6.205	17.96	0.8	-	Bushuyev et al. (2013)
[Zn (ata) (OH)]	2.54	54.1	-33.60	325	2.55	8.628	39.67	>40	>360	Su et al. (2017)
[Cu(atrz)_3_(NO_3_)_2_]_*n*_	1.680	53.35	-58.83	225	15.14	9.160	35.68	22.5	-	Li et al., 2013
NHP	1.983	33.49	-11.48	220	5.73	9.184	39.69	-	-	Bushuyev et al. (2012)
[Cu_4_Na(Mtta)_5_(CH_3_CN)]_*n*_	1.975	40.08	-71.97	335	9.90	7.225	24.43	36	>360	Feng et al. (2015)
[K_2_Zn(bta)_24_]_*n*_	2.015	48.69	-32.44	349	-	-	-	>40	>360	Li F. et al. (2015)
[Pb(bta)·2H_2_O]_*n*_	3.250	31.98	-22.32	330	4.97	8.963	47.47	>40	-	Liu J. et al. (2016)
[Cu_2_(to) (dns)]_*n*_	2.458	15.42	-47.55	217	4.10	7.522	29.51	38.6	>360	Yang et al., 2016
KCPT	1.98	39	-27.24	323	5.97	8.457	32.5	7.5	240	Li et al. (2017)
[Cu(Htztr)_2_(H_2_O)_2_]_*n*_	1.892	52.72	-60.24	345	-	8.18	30.57	>40	>360	Liu et al., 2015a
GO-TAG-Cu(II)	2.59	16.8	-68.6	451.5	-	7.815	26.7	>98	>360	Yan et al., 2016a
GO-TAG-Cu(II)	3.12	10.08	-80.6	495.5	-	7.723	23.2	81.3	>360	Yan et al., 2016a
TNT	1.654	18.50	-74.0	295	3.75	7.303	21.30	15.0	353	Su et al. (2017)
RDX	1.806	37.84	-21.61	205	5.80	8.795	34.90	7.5	120	Su et al. (2017)
HMX	1.950	37.80	-21.60	280	5.52	8.900	38.39	7.0	112	Su et al. (2017)
CL-20	2.04	38.36	0	221	6.16	9.730	44.4	4	48	Su et al. (2017)
DAP-4	1.87	9.76	-5.6	404	5.691	9.588	38.5	23	36	Chen et al., 2018a

a Density from X-ray diffraction analysis.

b Nitrogen content.

c Oxygen balance.

d Temperature of decomposition by DSC.

e Heat of detonation.

f Detonation velocity.

g Detonation pressure.

h Impact sensitivity.

i Friction sensitivity.

**FIGURE 14:**
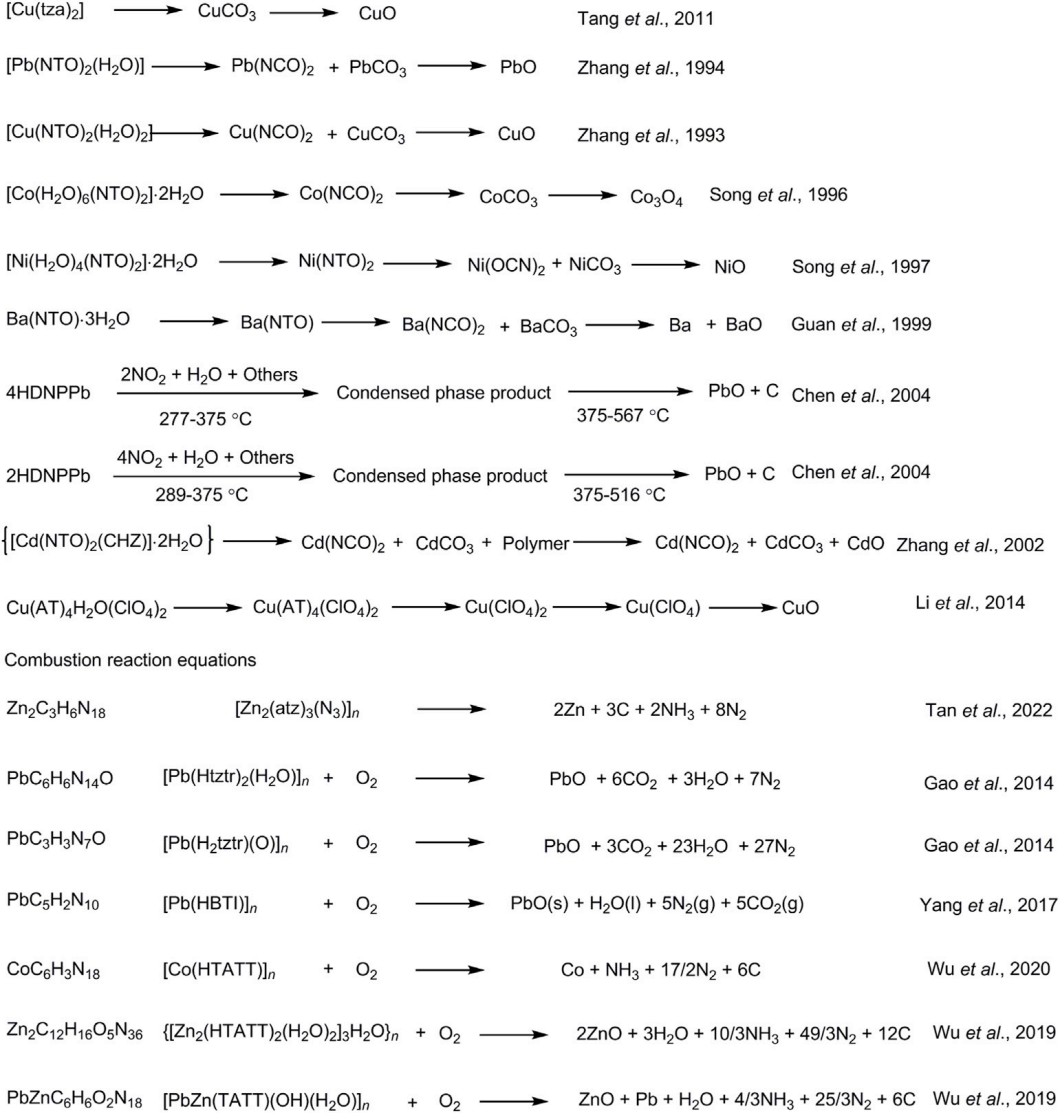
Diagram of catalytic combustion mechanism of typical compounds.

We can use various precise synthesis methods to fine-regulate the molecular composition and pore structure of high-energy EMOFs, design and synthesize solvent-free high-energy EMOFs, and obtain energetical burning rates catalysts with different energy and safety characteristics to meet the application requirements of different fields of solid propellant (Table 4). In addition, We should launch EMOFs-based burning rate catalyst structure-activity relationship studies, reveal the various structural parameters affect the performance of solid propellant combustion rule, from the angle of the catalytic mechanism of high efficient EMOFs guide the design and application of burning rate catalysts (Figure 14) (Ren et al., 2022; Luo, 2021; Zuo et al., 2021).

**TABLE 4:** Effect of different burning rate catalyst on burning performance of double base propellant.

Catalyst	Catalytic effect of burning	Platform area	Pressure index of platform area	Ref
Ba-NTO	Super-rate burning under 5 MPa, and the pressure exponent decreases significantly	none	0.389	Guan et al. (1999)
Pb(NTO)_2_	The burning rate increases obviously at 1 MPa, and the pressure exponent decreases at 10–20 MPa	none		Huang et al. (2013)
PbTMT	The burning rate increases obviously at 6 MPa, and the pressure exponent decreases at 10–14 MPa	none		Zhao et al. (2004)
CuTMT	The burning rate increases not obviously at 2–18 MPa, and the pressure exponent decreases at 8–14 MPa	none		Zhao et al. (2004)
SrTMT	The burning rate increases obviously at 4–6 MPa, and the pressure exponent increases at 6–18 MPa	none		Zhao et al. (2004)
CuTMT/*β*Pb	The burning rate increases obviously at 4 MPa, and the pressure exponent decreases at 8–12 MPa	8–12 MPa	-0.03	Zhao et al. (2004)
CuPHT	The burning rate increases obviously at 6 MPa, and the pressure exponent decreases at 8–12 MPa	none		Zhao et al. (2004)
CuPHT/*β*Pb	The burning rate increases obviously at 4 MPa, and the pressure exponent decreases at 10–18 MPa	10–18 MPa	0.295	Zhao et al. (2004)
PDNI	The burning rate increases obviously at 10 MPa, and the pressure exponent decreases at 18–22 MPa	12–18 MPa	0.15	Zheng et al. (2006)
PDNAP	Super-rate burning at 2–6 MPa, and the pressure exponent decreases at 6–10 MPa	6–10 MPa	0.24	Wang C. et al. (2012)
ANPyO-Co	Super-rate burning under 10 MPa, and the pressure exponent decreases at 6–10 MPa	none		Liu Q. et al. (2016)
BLS	Super-rate burning at 3.43–8.82 MPa, and the pressure exponent decreases at 10–14 MPa	4.89–6.86 MPa	0.18	Pundlik et al. (2001)
DNAS-Cu	Super-rate burning at 3.43–8.82 MPa, and the pressure exponent decreases at 3.43–4.89 MPa	3.43–4.89 MPa	0.24	Pundlik et al. (2001)
DNAS-Pb	Super-rate burning under 6 MPa, and the pressure exponent decreases significantly	6–10 MPa	0.13	Song et al. (2007)
Zr (3-NO_2_-PHT)_2_	The burning rate increases obviously at 2–6 MPa, and the pressure exponent decreases at 16–20 MPa	none		Zhang et al. (2009)
Zr (3-NO_2_-PHT)_2_/copper salts	The burning rate increases obviously at 6 MPa, and the pressure exponent decreases at 16–20 MPa	16–20 MPa	0.18	Zhang et al. (2009)
NP/β-Cu/CB	The burning rate increases obviously at 6 MPa, and the pressure exponent decreases at 10–16 MPa	10–16 MPa	0.221	Chang et al. (2008)
None	—	none		Chang et al. (2008)
NP/NC/CB	The burning rate increases obviously at 8 MPa, and the pressure exponent decreases at 10–16 MPa	10–16 MPa	0.165	Chang et al. (2008)
4-Hydroxy-3,5- dinitropyridine lead salt	Super-rate burning under 8 MPa, and the pressure exponent decreases significantly	none		Gao et al. (2012)
Cu(NTO)_2_	Super-rate burning at 7–13 MPa, increase the burning rate and decrease the pressure exponent	none		Zhang J. et al. (2020)
PbNI	Super-rate burning at 10–13 MPa, increase the burning rate and decrease the pressure exponent	none		Zhang W. et al. (2020)
Cu(NTO)_2_/PbNI	The burning rate increases obviously at 7 MPa, and the pressure exponent decreases at 7–13 MPa	7–10 MPa	0.27	Zhang J. et al. (2020)
Cu(NTO)_2_/PbNI/CB	The burning rate increases obviously at 7 MPa, and the pressure exponent decreases at 7–13 MPa	7–10 MPa	0.18	Zhang W. et al. (2020)

## Summary and outlook

In this paper, EMOFs-based burning rate catalysts for solid propellants are explored from the following four aspects. First, the design and preparation of a new multifunctional EMOFs burning rate catalyst are investigated, and its structure and stability are characterized. Second, the compatibility and stability of the new burning rate catalyst and propellant components are evaluated. Third, the kinetics and mechanism of thermal decomposition of propellant components catalyzed by burning rate catalysts are analyzed. Fourth, the changes of the burning rate pressure index, flame structure and combustion wave temperature of solid propellants under catalytic conditions are discussed. However, EMOFs-based burning rate catalysts are not applicable to the catalysis of all propellants. In other words, different types of catalysts exhibit good catalytic effects on specific types of propellants. Besides, the catalytic effect of the same catalyst varies in different burning systems. The type and structure of the catalyst are the main factors affecting its catalytic effect.

To develop EMOFs-based energetic burning rate catalysts (for solid propellants) that can satisfy the performance requirements of future weapons, future research should focus on environmental protection, high energy and low sensitivity, nanometerization, multifunctional composites and solvent-free.

1) Environmental protection: EMOFs-based burning rate catalysts contain heavy metals such as lead, which will pollute the environment. It has become an inevitable trend to develop green burning rate catalysts, such as bismuth-containing catalysts with low toxicity, less smoke emissions and ecological safety. Green and environmentally friendly EMOFs catalysts and high-efficiency bimetallic composite catalysts are also the priority of future research on green burning rate catalysts.

2) High energy and low sensitivity: Burning rate catalysts containing energy can reduce the energy loss of the solid propellant. Researchers are committed to developing EMOFs-based burning rate catalysts with both high energy and low sensitivity. However, it is a difficulty to simultaneously achieve high energy and low inductance in energetic catalysts.

3) Nanometerization: Nanometerization has always been an effective way to improve the catalytic activity of energetic burning rate catalysts. Nano-composites have the advantages of small particle size, large specific surface area, good dispersion and high catalytic activity. Nano-composites can greatly improve the catalytic performance of burning rate catalysts and reduce the quantity of catalysts used. In recent years, nano-EMOFs-based burning rate catalysts loaded on carbon substrates have drawn attention and have been gradually applied to propellants.

4) Multifunctional composites: With the increasingly complex composition of energetic materials and the continuous development of new single-element high-energy materials, traditional EMOFs-based burning rate catalysts with a single catalytic function can no longer fully meet the application requirements. Combining EMOFs with organic/inorganic composite catalysts and multimetallic synergistic catalysts, and grafting EMOFs into binders can diversify and modify the catalytic effects on solid propellants. In the future, it will be an important development direction to diversify the catalytic role of catalysts in propellant combustion through bonding, viscosity reduction, and process improvement.

5) Solvent-free: At present, the synthesis of high-energy EMOFs is mostly carried out in solution. In the process of synthesis, solvent molecules with coordination ability, like other ligands, can also coordinate with the metal center, occupy the lattice of EMOFs, or exist in the pore structure of EMOFs, and become the component of EMOFs. On the one hand, the presence of non-energetic solvent molecules will reduce the energy density of high-energy EMOFs, which is not conducive to the improvement of their energy level. On the other hand, under certain temperature conditions, solvent molecules are easy to detangle from the structure of EMOFs, which reduces the structural rigidity of MOFs and results in the decline of the stability of EMOFs. The existing research results showed that the sensitivity of solvent-free high energy EMOFs is usually low. Therefore, the synthesis of high energy EMOFs without solvent is also an important means to improve their energy and safety performance.
